# Biting midges of Egypt (Diptera: Ceratopogonidae)

**DOI:** 10.3897/BDJ.8.e52357

**Published:** 2020-04-29

**Authors:** Magdi S. El-Hawagry, Salah El-Din A. El-Azab, Mahmoud S. Abdel-Dayem, Hathal M. Al Dhafer

**Affiliations:** 1 Entomology Department, Faculty of Science, Cairo University, Giza, Egypt Entomology Department, Faculty of Science, Cairo University Giza Egypt; 2 Insect Taxonomy Department, Plant Protection Research Institute, Dokki, Giza, Egypt Insect Taxonomy Department, Plant Protection Research Institute, Dokki Giza Egypt; 3 College of Food and Agricultural Sciences, King Saud University, Riyadh, Saudi Arabia College of Food and Agricultural Sciences, King Saud University Riyadh Saudi Arabia

**Keywords:** Checklist, geographical distribution, Egyptian localities, dates of collection.

## Abstract

**Background:**

This study is one in a series of planned studies on different Egyptian dipteran taxa aiming to catalogue the whole order in Egypt.

**New information:**

All known Egyptian taxa of the family Ceratopogonidae (biting midges) are systematically catalogued. A total number of 64 species belonging to 11 genera, four tribes and four subfamilies has been treated. Data for this study have been compiled from both available literature and specimens collected from different Egyptian localities by the authors. An updated classification, synonymies, type localities, world distributions by biogeographic realm(s) and country, Egyptian localities and dates of collection are provided comprising some new locality records. The study treats all previous inaccuracies in the classification of the family in Egypt.

## Introduction

Ceratopogonidae is an extremely diverse family of small nematocerous flies (usually less than 3 mm) commonly known as biting midges, with some 130 recognised genera and almost 6,300 extant species distributed worldwide (Pape et al. 2011, Borkent 2016). The family includes also numerous fossils from the Lower Cretaceous to the Miocene, with over 280 species representing 49 genera (26 extant and 23 extinct) (Szadziewski 2018).

The females of most ceratopogonid species are blood-suckers of vertebrates, particularly along sea shores, rivers, lakes and mangrove swamps. Members of the genera *Culicoides* Latreille and *Leptoconops* Skuse are the best known to suck the blood of vertebrate animals, including humans, to get protein for egg-laying. *Culicoides* is the most important ever, as many species of which are implicated in the spread of pathogens and parasites to man and other animals. However, although members of the genus *Leptoconops* are not known to be involved in the spread of disease, they are important biting pests as their bites are painful and reactions to them last for several days in sensitive people (Linley and Davies 1971, Downes 1978). Identification of the vector and information on the major vector species are important steps in the epidemiology of vector-borne diseases. This will give a clearer indication of the geographic distribution of the disease or its potential distribution (Rawlings 1996). Some species of the genus *Forcipomyia* Meigen are nectar-feeders and are of great economic importance as pollinators of tropical crop plants (Downes 1978, Wilkening et al. 1985).

Ceratopogonid adults can be distinguished by their wings: wing with hind margin with simple setulae, with eight or fewer longitudinal veins reaching margin, vein M_1+2_ usually forked, vein R_2+3_ straight or virtually so (arrowed) not arched and basal medial cross-vein absent (Kirk-Spriggs and Sinclair 2017). The larvae of *Culicoides* are slender and nematode-like, without appendages and are usually whitish with occasional thoracic pigment patterns. They are aquatic and swim with a characteristic eel-like motion. The larvae of *Leptoconops* are semi-aquatic. In contrast, the terrestrial larvae of Forcipomyia subgenus Lasiohelea are found in sandy soil, under rotting bark or in damp moss. The pupae are usually hidden in litter or mud, amongst floating vegetation or, in the case of *Lasiohelea*, under moss or rotting bark. Those of *Leptoconops* species found on beaches occur in the sand at or near the high-water mark (Boorman 1993).

In Egypt, biting midges constitute a major threat to the tourism industry, especially at recreational beaches and in the resort areas of Southern Sinai, the North Coast and the Red Sea shores (Ghonaim et al. 2001b). This catalogue will hopefully be a base for future studies on monitoring of Egyptian biting midges as well as increasing the knowledge of their geographic range (Rawlings 1996).

Some previous studies have been carried out to study the Egyptian fauna of biting midges, including: Becker (1903), Kieffer (1908), Kieffer (1925c), Macfie (1924), Macfie (1943), Macfie (1944), Nagaty and Morsy (1959a), Nagaty and Morsy (1959b), Nagaty and Morsy (1960a), Nagaty and Morsy (1960b), Morsy (1961), Steyskal and El-Bialy (1967), Abdel-Samei (1974), Eesa (1975), Morsy et al. (1989), Braverman et al. (1981), Ahmed (1991), Ghonaim (1997), Ghonaim et al. (2001a). From our viewpoint, Kieffer (1925c) and Macfie (1943), in particular, were the most comprehensive studies. Kieffer (1925c) collected 22 ceratopogonid species from different localities in the Coastal Strip, Eastern Desert, Lower Nile Valley & Delta and Upper Nile Valley. He collected these species abundantly from the Coastal Strip and Eastern Desert in April and from Lower Nile Valley & Delta and Upper Nile Valley in October and November. On the other hand, Macfie (1943) collected 23 different ceratopogonid species from Moascar, near Ismailia (Eastern Desert). He collected most of these species in the evening on windows in February, as they were fairly numerous, in March and April, as they were abundant, but in May they again became fewer and, by the middle of that month, were scarce or had entirely vanished. Macfie observed that they were particularly abundant after dust storms, as if they had sought shelter in buildings from the hot, dry, dust-laden wind.

Ghonaim et al. (2001b) summarised all previous studies and presented a checklist of all Egyptian Ceratopogonidae; however, they wrongly listed *Alluaudomyia vudu* De Meillon and Hardy, 1954, *Culicoides arenarius* Edwards, 1922, *Culicoides inornatipennis* Carter, Ingram & Macfie, 1920 and *Dasyhelea inconspicuosa* Carter, Ingram and Macfie, 1921 as recorded from Egypt by Macfie (1943). In fact, Macfie (1943) did not record any of these species from Egypt and this confusion might result from a misconception of the application of the term variety used by Macfie or a misinterpretation of some of his discussions. In addition, the list of Ghonaim et al. (2001b) included an inaccuracy for the application of the term *Nomen dubium* for the species *Atrichopogon isis* Kieffer, *Culicoides arenarius* Edwards, *Dasyhelea egypti* Macfie, *Dasyhelea heliophila* Macfie and *Dasyhelea inconspicuosa* Carter, Ingram and Macfie.

In the present study, a total of 64 species belonging to 11 genera, four tribes and four subfamilies has been catalogued (Table 1) and all previous inaccuracies are treated.

This study is one in a series of planned studies on different Egyptian dipteran taxa aiming to catalogue the whole order in Egypt (EL-Hawagry 2015, El-Hawagry 2017, El-Hawagry 2018, El-Hawagry et al. 2018, El-Hawagry and El-Azab 2019, El-Hawagry et al. 2019, El-Hawagry and Gilbert 2019).

## Materials and methods

**Data sources**. The present catalogue is based mainly on data obtained from previous studies on the Egyptian Ceratopogonidae (all treated species have been recorded in previous studies), in addition to specimens collected by the author and his co-workers from different Egyptian localities using light traps (only 8 species: *Atrichopogon homoius* Ingram and Macfie, *Atrichopogon luteicollis* (Becker), *Forcipomyia psilonota* (Kieffer), *Forcipomyia fuliginosa* (Meigen), *Culicoides circumscriptus* Kieffer, *Culicoides distinctipennis* Austen, *Culicoides puncticollis* (Becker) and *Culicoides schultzei* (Enderlein)). A great deal of information, including synonymies (particularly Old World synonyms) and distributional data has been obtained from relevant literature and website databases as well. These sources are listed in the following subsections.

**Figure 1.**
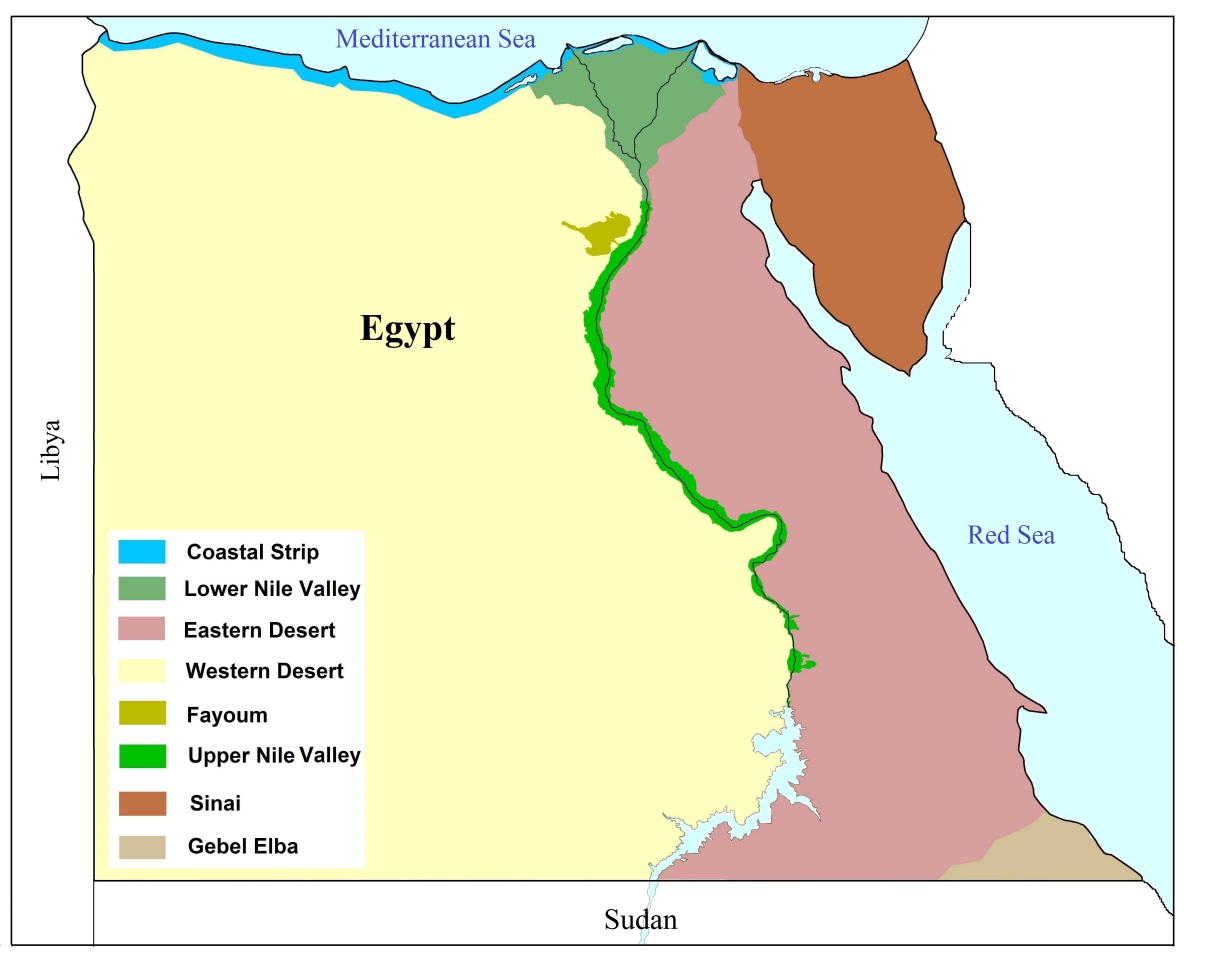
Map of Egypt showing the ecological zones

**Study area**. Egypt, the study area, is a part of the Great Desert Belt, it is characterised by a warm and almost rainless climate and it is one of the driest countries in the world (FAO 2012, Nasser et al. 2019). It is divided into eight ecological zones: the Coastal Strip, Lower Nile Valley & Delta, Upper Nile Valley, Fayoum, Eastern Desert, Western Desert, Sinai and Gebel Elba (Fig. 1). The fauna of all but one of these zones is mostly affiliated to the Palaearctic Region, but that of Gebel Elba, the south-eastern triangle of Egypt, has a greater affiliation to the Afrotropical Region (El-Hawagry and Gilbert 2014, El-Hawagry et al. 2020).

**Classification and arrangement of taxa.** The current classification and arrangement of taxa of the family Ceratopogonidae basically follows that used in the world catalogues (Borkent 2016, Borkent and Wirth 1997), in which the family is divided into four subfamilies: Leptoconopinae, Forcipomyiinae, Dasyheleinae and Ceratopogoninae. The Ceratopogoninae is the largest Egyptian subfamily, including four tribes in Egypt, namely Culicoidini, Ceratopogonini, Johannsenomyiini and Palpomyiini.

**World distribution**. Data on world distribution are mainly according to Remm (1988), Systema Dipterorum (http://sd.zoobank.org), Filatov and Szadziewski (2017), Dominiak and Szadziewski (2010), Alwin-Kownacka et al. (2016) and Alwin-Kownacka et al. (2017).

**Local distribution (Egyptian localities) and dates of collection**. The distributional data and dates of collection that are known so far in the different ecological zones of Egypt are given. Localities within each ecological zone are arranged alphabetically and written after a colon following the ecological zone, for example, "Lower Nile Valley & Delta: EI-Qanatir, EI-Mansoura, Marsafa ".

The basic sources for this part of the catalogue are data from some specimens collected from different Egyptian localities by the authors, in addition to literature records from previous studies on the Egyptian fauna of biting midges, including: Becker (1903), Kieffer (1908), Kieffer (1925c), Macfie (1924), Macfie (1943), Macfie (1944), Nagaty and Morsy (1959a), Nagaty and Morsy (1959b), Nagaty and Morsy (1960a), Nagaty and Morsy (1960b), Morsy (1961), Steyskal and El-Bialy (1967), Abdel-Samei (1974), Eesa (1975), Morsy et al. (1989), Braverman et al. (1981), Ahmed (1991), Ghonaim (1997), Ghonaim et al. (2001a). If the Egyptian localities or dates of collection of adult were not known, the term “Unknown” is used.

Abbreviations used:

- AF, Afrotropical
- AU, Australasian
- ICZN, The International Commission of Zoological Nomenclature
- Is., Islands
- N., North
- NE, Nearctic
- NT, Neotropical
- OR, Oriental
- PA, Palaearctic
- S., South
- St., Saint
- USA, United States of America
- var., variety

## Checklists

### Catalogue of the Ceratopogonidae of Egypt

#### LEPTOCONOPINAE

1020E87A-A5E9-54C1-BE3D-1AAAE6F28ABA

#### Leptoconops

Skuse, 1889

1E6BB6AB-AE03-50F8-9B45-F932B22A53E6

https://www.gbif.org/species/1633120

Leptoconops ***Leptoconops*** Skuse, 1889 [Skuse 1889: 288]. Type species: *Leptoconops stygius* Skuse, by monotypy.
Mycterotypus Noè, 1905 [Noè 1905: 114]. Type species: *Mycterotypus bezzii* Noè, by designation of Carter (1921).
Protersesthes Kieffer, 1921 [*Kieffer 1921b*: 107]. Type species: *Tersesthes brasiliensis* Lutz, by original designation.
Tersesthes Townsend, 1893 [*Townsend 1893*: 370]. Type species: *Tersesthes torrens* Townsend, by original designation.
Schizoconops Kieffer, 1918 [Kieffer 1918a: 135]. Type species: *Schizoconops indicus* Kieffer, by monotypy.

#### Holoconops

Kieffer, 1918

6F07BE06-E249-5479-861D-FD3B5F26C245

Holoconops ***Holoconops*** Kieffer, 1918 [Kieffer 1918a]: 135. Type species: *Leptoconops kerteszi* Kieffer, by original designation.
Microconops Kieffer, 1921 [*Kieffer 1921b*: 108]. Type species: *Microconops vexans* Kieffer, by original designation.

#### Leptoconops (Holoconops) kerteszi

Kieffer, 1908

DDB48363-3CB5-5734-BBCB-97FF2DA6C282

Leptoconops kerteszi Kieffer, 1908 [Kieffer 1908: 576]. Type locality: Egypt (Cairo).

##### Distribution

PA: Egypt.

Local distribution in Egypt: Eastern Desert: Ismailia (Gebel Maryiam, Moaskar). Lower Nile Valley & Delta: Cairo. Sinai: Bir El-Abd. Western Desert: Saqqara, Wadi El-Natroun.

Dates of collection in Egypt: December to July.

#### Leptoconops (Holoconops) macfiei

Clastrier, 1975

91796C1D-FE3B-5BD8-9FC9-54CD87968C83

Leptoconops macfiei Clastrier, 1975 [Clastrier 1975: 28]. Type locality: Egypt (Ismailia).

##### Distribution

AF: Sudan, Yemen (Kamaran Is.). PA: Egypt.

Local distribution in Egypt: Eastern Desert: Ismailia (Gebel Maryiam, Moaskar). Sinai: Bir El-Abd.

Dates of collection in Egypt: January to April.

#### Leptoconops (Holoconops) transversalis

(Kieffer, 1921)

B2197B89-20B5-5F29-9E3C-E536ED538F1D

Holoconops transversalis Kieffer, 1921 [*Kieffer 1921b*: 112]. Type locality: Tunisia.

##### Distribution

PA: ?Egypt, Tunisia.

Local distribution in Egypt: Unknown.

Dates of collection in Egypt: Unknown.

##### Notes

?- This species was listed as recorded from Egypt by Steyskal & El-Bialy (1967), but no specimens or published records have been found

#### FORCIPOMYIINAE

97D87AD0-D3D2-5216-BD44-47F51EADC3B3

#### Atrichopogon

Kieffer, 1906

08337CB5-1D63-5A11-980E-E26C8AB5E008

https://www.gbif.org/species/1636637

Atrichopogon ***Atrichopogon*** Kieffer, 1906 [Kieffer 1906: 53]. Type species: *Ceratopogon exilis* Coquillett, by subsequent designation of Coquillett (1910).
Kempia Kieffer, 1913 [*Kieffer 1913c*: 162, 179]. Type species: *Dasyhelea calcuttensis* Kieffer, by subsequent designation of Wirth (1952).
Gymnohelea Kieffer, 1921 [*Kieffer 1921b*: 115]. Type species: *Kempia longiserrus* Kieffer, by subsequent designation of Wirth (1973).
Dolichohelea Edwards, 1929 [*Edwards 1929*: 8]. Type species: *Dolichohelea polita* Edwards, by monotypy.
Lophomyidium Cordero, 1929 [*Cordero 1929*: 94]. Type species: *Lophomyidium uruguayense* Cordero, by original designation.
Psilokempia Enderlein, 1936 [Enderlein 1936: 49]. Type species: *Kempia appendiculata* Goetghebuer, by monotypy.
Meloehelea Wirth, 1956 [*Wirth 1956*: 16]. Type species: *Atrichopogon meloesugans* Kieffer.
Psammopogon Remm, 1979 [*Remm 1979*: 57]. Type species: *Atrichopogon trifasciata* Kieffer, by original designation.
Rostropogon Remm, 1979 [*Remm 1979*: 57]. Type species: *Ceratopogon rostratus* Winnertz, by original designation.
Impensukempia Yu, 2001 [Liu et al. 2001: 47]. Type species: *Atrichopogon impensus* Yu and Yan, by original designation.
Bessamyia Yu, Liu, Liu, Liu, Hao, Yan and Zhao, 2005 [*Yu et al. 2005*: 355]. Type species: *Atrichopogon bessa* Yu and Yan, by original designation.

#### Atrichopogon callipotami

Macfie, 1924

750559C5-A521-5D8A-ADBD-AA7C22C23F18

Atrichopogon callipotami Macfie, 1924 [Macfie 1924: 65]. Type locality: Egypt (Nile at Cairo).

##### Distribution

PA: Egypt.

Local distribution in Egypt: Lower Nile Valley & Delta: Cairo.

Dates of collection in Egypt: December.

#### Atrichopogon flavitarsatus

(Becker, 1903)

086C69B5-3C61-503F-9E7C-D6F123FA3F42

Ceratopogon flavitarsatus Becker, 1903 [Becker 1903: 74]. Type locality: Egypt (Luxor).
Atrichopogon flavitarsatus : Szadziewski, 1984 [Szadziewski 1984: 187].

##### Distribution

PA: Egypt, Spain.

Local distribution in Egypt: Upper Nile Valley: Luxor.

Dates of collection in Egypt: December.

#### Atrichopogon homoius

Ingram and Macfie, 1921

8E74F7F5-F929-5A8B-BE92-6E26751EFF8A

Atrichopogon homoium Ingram & Macfie, 1921 [Ingram and Macfie 1921: 338]. Type locality: Ghana.
Atrichopogon alfierii Kieffer, 1925 [*Kieffer 1925c*: 249]. Type locality: Egypt.

##### Materials

- **Type status:** Other material. **Occurrence:** recordNumber: 1; sex: male; lifeStage: adult; disposition: personalcollection of El-Hawagry; **Taxon:** scientificName: *Atrichopogon homoius* Ingram and Macfie, 1921; kingdom: Animalia; phylum: Arthropoda; class: Insecta; order: Diptera; family: Ceratopogonidae; **Location:** continent: Africa; country: Egypt; stateProvince: Giza; locality: El-Hager; **Event:** samplingProtocol: light trap; eventDate: 15 Aug 1996

##### Distribution

AF: Ghana. PA: Egypt.

Local distribution in Egypt: Lower Nile Valley & Delta: Ayiat, El-Hager (Mansheyet El-Qanatir), El-Qanatir, El-Badrasheen, Maadi. Upper Nile Valley: Assiout, Asswan, Beni Hassan, Dendera, Girga.

Dates of collection in Egypt: August to December.

#### Atrichopogon isis

Kieffer, 1925

586E097B-0BFB-5F17-93F7-BBBFBC896DC9

Atrichopogon isis Kieffer, 1925 [*Kieffer 1925c*: 252]. Type locality: Egypt (Maadi).

##### Distribution

PA: Egypt.

Local distribution in Egypt: Lower Nile Valley & Delta: Maadi (at border of the Nile).

Dates of collection in Egypt: October.

##### Notes

Alwin-Kownacka et al. (2016) treated this species as a nomen dubium because it is poorly described and types most probably not preserved.

#### Atrichopogon luteicollis

(Becker, 1903)

EB90C672-44E2-522A-A8C6-4A61E51851D0

Ceratopogon luteicollis Becker, 1903 [Becker 1903: 74]. Type locality: Egypt (Asswan).
Ceratopogon flavoscutellatus Becker, 1908 [Becker 1908: 74] (preoccupied by *Dasyhelea flavoscutellata* (Zetterstedt 1850). Type locality: Canary Islands (Spain).
Atrichopogon atriscapula : Macfie 1924 [Macfie 1924: 63], (= *Atrichopogon atriscapulus* Kieffer, 1918), nec Kieffer 1918b: 45.
Atrichopogon aegyptius Kieffer, 1925 [*Kieffer 1925c*: 250]. Type locality: Egypt.
Atrichopogon phrixus de Meillon, 1943 [De Meillon 1943: 105]. Type locality: South Africa.
Atrichopogon sanani Boorman & van Harten, 2002 [*Boorman and van Harten 2002*: 434]. Type locality: Yemen.

##### Materials

- **Type status:** Other material. **Occurrence:** individualCount: 1; sex: male; lifeStage: adult; disposition: personal collection of El-Hawagry; **Taxon:** scientificName: *Atrichopogon luteicollis* (Becker, 1903); kingdom: Animalia; phylum: Arthropoda; class: Insecta; order: Diptera; family: Ceratopogonidae; **Location:** continent: Africa; country: Egypt; stateProvince: Giza; locality: El-Hager; **Event:** eventDate: 15 Aug 1996

##### Distribution

AF: South Africa, Sudan, Yemen. PA: Algeria, Canary Islands, Egypt, Israel, Spain.

Local distribution in Egypt: Fayoum: Shakshook. Lower Nile Valley & Delta: Cairo, El-Hager (Mansheyet El-Qanatir), Maadi (at border of the Nile). Upper Nile Valley: Asswan, Beni Hassan, Dendera, Edfu, Girga, Luxor. El Sammaneen (Luxor). Western Desert: El-Bustan.

Dates of collection in Egypt: August to December.

#### Atrichopogon osiris

Kieffer, 1925

05B66C9C-5BAE-5214-9794-F876ADA118AB

Atrichopogon osiris Kieffer, 1925 [*Kieffer 1925c*: 250]. Type locality: Egypt (Maadi).

##### Distribution

PA: Egypt.

Local distribution in Egypt: Lower Nile Valley & Delta: Maadi.

Dates of collection in Egypt: October.

##### Notes

Alwin-Kownacka et al. (2016) treated this species as a *nomen dubium* because it is poorly described and types most probably not preserved

#### Forcipomyia

Meigen, 1818

F47B53EC-96F7-58E4-86A8-636AEC76524C

https://www.gbif.org/species/1633624

Forcipomyia ***Forcipomyia*** Meigen, 1818 [Meigen 1818: 73, 75]. Type species: *Tipula bipunctata* Linnaeus, by subsequent designation of Westwood (1840).
Labidomyia Stephens, 1829 [*Stephens 1829a*: 52 (1829b: 239)]. Type species: *Tipula bipunctata* Linnaeus, by subsequent designation of Westwood (1840).
Tetraphora Philippi, 1865 [*Philippi 1865*: 630]. Type species: *Tetraphora fusca* Philippi, by monotypy.
Prohelea Kieffer, 1911 [*Kieffer 1911a*: 319]. Type species: *Ceratopogon decipiens* Kieffer, by subsequent designation of Brunetti (1920).

#### Euprojoannisia

Brèthes, 1914

94D149D7-B930-5685-B85F-9BADADBB42D2

Euprojoannisia ***Euprojoannisia*** Brèthes, 1914 [Brèthes 1914: 155]. Type species: *Euprojoannisia platensis* Brèthes, by original designation.
Euforcipomyia Malloch, 1915 [Malloch 1915b: 312]. Type species: *Euforcipomyia hirtipennis* Malloch (= *Ceratopogon palustris* Meigen), by original designation.
Cryptoscena Enderlein, 1936 [*Enderlein 1936*: 51]. Type species: *Ceratopogon palustris* Meigen, by monotypy.
Proforcipomyia Saunders, 1957 [Saunders 1957: 662]. Type species: *Forcipomyia wirthi* Saunders, by original designation.

#### Forcipomyia (Euprojoannisia) psilonota

(Kieffer, 1911)

28BDA2C6-AEAB-5C46-9238-5F3E1810499F

Ceratopogon psilonota Kieffer, 1911 [Kieffer 1911b: 337]. Type locality: Seychelles.
Ceratopogon aplonota Kieffer, 1911 [Kieffer 1911b: 337]. Type locality: Seychelles.
Ceratopogon seychelleana Kieffer, 1911 [Kieffer 1911b: 338]. Type locality: Seychelles.
Ceratopogon seychelleana var. fulvithorax Kieffer, 1911 [Kieffer 1911b: 338]. Type locality: Seychelles.
Forcipomyia indecora Kieffer, 1914 [Kieffer 1914b: 269]. Type locality: South Africa.
Forcipomyia litoralis Santos Abreu, 1918 [Santos Abreu 1918: 277]. Type locality: Canary Islands (Spain).
Forcipomyia ingrami Carter, 1919 [Carter 1919: 290]. Type locality: Ghana.
Forcipomyia egypti Macfie, 1924 [Macfie 1924: 61]. Type locality: Egypt (Nile near Ayiat).
Forcipomyia hathor Kieffer, 1925 [*Kieffer 1925c*: 247]. Type locality: Egypt (Maadi).
Forcipomyia conogensis Goetghebuer, 1933 [Goetghebuer 1933b: 132]. Type locality: Democratic Republic of the Congo.
Forcipomyia flavipilosella Goetghebuer, 1933 [Goetghebuer 1933b: 130]. Type locality: Democratic Republic of the Congo.
Forcipomyia lulengaensis Goetghebuer, 1935 [Goetghebuer 1935: 155]. Type locality: Democratic Republic of the Congo.
Forcipomyia superata Goetghebuer, 1935 [Goetghebuer 1935: 160]. Type locality: Democratic Republic of the Congo.
Forcipomyia griseipluma Goetghebuer, 1935 [Goetghebuer 1935: 154]. Type locality: Democratic Republic of the Congo.
Forcipomyia griseolella Goetghebuer, 1948 [Goetghebuer 1948: 7]. Type locality: Democratic Republic of the Congo.

##### Materials

- **Type status:** Other material. **Occurrence:** individualCount: 2; sex: female; lifeStage: adult; disposition: personal collection of El-Hawagry; **Taxon:** scientificName: *Forcipomyia psilonota* (Kieffer, 1911); kingdom: Animalia; phylum: Arthropoda; class: Insecta; order: Diptera; family: Ceratopogonidae; **Location:** continent: Africa; country: Egypt; stateProvince: Asswan; locality: Asswan; **Event:** samplingProtocol: light trap; eventDate: 17 Dec 1998

##### Distribution

AF: Widely distributed. PA: Algeria, Andorra, Azores, Bahrain, Canary Is., Egypt, Israel, Spain.

Local distribution in Egypt: Eastern Desert: Ismailia (Moaskar). Lower Nile Valley & Delta: Ayiat, El-Qanatir, Maadi. Upper Nile Valley: Asswan, Benni Hassan. Western Desert: El-Bustan.

Dates of collection in Egypt: August to December.

#### Forcipomyia

Meigen, 1818 —Nominotypical subgenus

AA4BCF57-5C54-528F-A1F3-DA62CB8731FA

#### Forcipomyia (Forcipomyia) biannulata

Ingram and Macfie, 1924

AE8504F0-084B-5813-B0E8-60146BD36722

Forcipomyia biannulata Ingram & Macfie, 1924 [Ingram and Macfie 1924: 557]. Type locality: Ghana, Nigeria, Malawi.
Forcipomyia bicolorata Goetghebuer, 1935 [Goetghebuer 1935: 150]. Type locality: Democratic Republic of the Congo.
Forcipomyia marginella Goetghebuer, 1935 [Goetghebuer 1935: 156]. Type locality: Democratic Republic of the Congo.
Forcipomyia quatuorguttata Goetghebuer, 1935 [Goetghebuer 1935: 158]. Type locality: Democratic Republic of the Congo.
Forcipomyia nigricosta Goetghebuer, 1935 [Goetghebuer 1935: 158]. Type locality: Democratic Republic of the Congo.
Forcipomyia pallidula Goetghebuer, 1948 [Goetghebuer 1948: 6]. Type locality: Democratic Republic of the Congo.
Forcipomyia abonnenci Clastrier, 1959 [*Clastrier 1959*: 340]. Type locality: Senegal.

##### Distribution

AF: Widespread. PA: Canary Islands, Egypt, Israel.

Local distribution in Egypt: Eastern Desert: Ismailia (Moaskar). Fayoum: Shakshook. Lower Nile Valley & Delta: El-Badrasheen, El-Qanatir. Upper Nile Valley: Asswan, Sohag (El-Shewash).

Dates of collection in Egypt: September to February.

#### Forcipomyia (Forcipomyia) nilicola

(Kieffer, 1925)

892FF8D5-9F37-554D-A535-FFC0DEE5CC81

Apelma nilicola Kieffer, 1925 [*Kieffer 1925c*: 253]. Type locality: Egypt (Maadi).

##### Distribution

PA: Egypt.

Local distribution in Egypt: Lower Nile Valley & Delta: Maadi. Upper Nile Valley: Sohag (El-Shewash).

Dates of collection in Egypt: September and October.

##### Notes

Alwin-Kownacka et al. (2016) treated this species as a *nomen dubium* because it is poorly described and types most probably not preserved.

#### Forcipomyia (Forcipomyia) urnigera

Kieffer, 1925

4B6F2086-4401-580F-88D7-063F71EC048B

Forcipomyia urnigera Kieffer, 1925 [*Kieffer 1925c*: 247]. Type locality: Egypt (Maadi).

##### Distribution

PA: Egypt.

Local distribution in Egypt: Lower Nile Valley & Delta: Maadi (at border of the Nile).

Dates of collection in Egypt: July.

##### Notes

Alwin-Kownacka et al. (2016) treated this species as a *nomen dubium* because it is poorly described and types most probably not preserved

#### Lepidohelea

Kieffer, 1917

BA356BF2-3926-5928-8374-11B1E99BD166

Lepidohelea ***Lepidohelea*** Kieffer, 1917 [Kieffer 1917: 364]. Type species: *Ceratopogon chrysolophus* Kieffer, by original designation.

#### Forcipomyia (Lepidohelea) pulcherrima

Santos Abreu, 1918

BB19C862-3439-5456-894E-AF7F15B95AF0

Forcipomyia pulcherrima Santos Abreu, 1918 [Santos Abreu 1918: 272]. Type locality: Canary Islands (Spain).
Lepidohelea ornatipes Kieffer, 1921 [Kieffer 1921a: 1], preoccupied by *Forcipomyia ornatipes* (Kieffer 1918a). Type locality: Cameroon.
Lepidohelea formosae Kieffer, 1922 [Kieffer 1922a: 153]. Type locality: Taiwan.
Forcipomyia lepidota Ingram and Macfie, 1924 [Ingram and Macfie 1924: 566]. Type locality: Ghana.
Forcipomyia variegata Goetghebuer, 1933 [Goetghebuer 1933b: 133]. Type locality: Democratic Republic of the Congo.
Forcipomyia marsafae Ghonaim, Ibrahim and Ali, 2001 [Ghonaim et al. 2001b: 42]. Type locality: Egypt (Qalyubiya: Marsafa).

##### Distribution

AF: Widely distributed. AU: Hawaii. NE: USA. OR: Widely distributed. PA: Canary Islands, Egypt, Israel, Japan, Lebanon, Saudi Arabia.

Local distribution in Egypt: Lower Nile Valley & Delta: El-Badrasheen, El-Qanatir, Marsafa. Upper Nile Valley: Asswan, Luxor (El-Sammaneen), Sohag (El-Shewash).

Dates of collection in Egypt: September to April.

#### Microhelea

Kieffer, 1917

90BC5950-DAA2-5554-BAB9-E6D69BF47423

Microhelea ***Microhelea*** Kieffer, 1917 [*Kieffer 1917*: 364]. Type species: *Atrichopogon microtomus* Kieffer, by subsequent designation of Kieffer (1921b: 7).
Phasmidohelea Mayer, 1937 [*Mayer 1937*: 233]. Type species: *Phasmidohelea crudelis* Mayer, by original designation.

#### Forcipomyia (Microhelea) fuliginosa

(Meigen, 1818)

B46B4920-F5A0-5FB4-806E-79902AB86DEE

Ceratopogon fuliginosa Meigen, 1818 [Meigen 1818: 86]. Type locality: Germany.
Ceratopogon villosa Zetterstedt, 1850 [Zetterstedt 1850: 3645]. Type locality: Sweden.
Ceratopogon crudelis Karsch, 1886 [Karsch 1886: 18]. Type locality: Germany.
Ceratopogon hirtipes de Meijere, 1907 [*De Meijere 1907*: 209]. Type locality: Indonesia.
Forcipomyia brevimana Lundström, 1910 [Lundström 1910: 32]. Type locality: Finland.
Ceratopogon inornatipennis Austen, 1912 [Austen 1912: 107]. Type locality: Nigeria.
Forcipomyia bipunctata var. obscura Santos Abreu, 1918 [Santos Abreu 1918: 276], preoccupied by *Forcipomyia obscura* (Walker 1848). Type locality: Canary Islands (Spain).
Ceratopogon alboclavata Kieffer, 1919 [Kieffer 1919: 12]. Type locality: Slovak Republic.
Ceratopogon canaliculata Goetghebuer, 1920 [Goetghebuer 1920: 110]. Type locality: Belgium.
Forcipomyia nilotheres Macfie, 1924 [Macfie 1924: 62]. Type locality: Egypt (Nile at Assiout).
Forcipomyia inornatipennis var. ornaticrus Ingram and Macfie, 1924 [Ingram and Macfie 1924: 577], preoccupied by *Forcipomyia ornaticrus*Kieffer 1912. Type locality: Ghana.
Forcipomyia atripennis Goetghebuer, 1935 [Goetghebuer 1935: 149]. Type locality: Democratic Republic of the Congo.
Forcipomyia auripila Goetghebuer, 1935 [Goetghebuer 1935: 150]. Type locality: Democratic Republic of the Congo.
Forcipomyia curtimana Goetghebuer, 1935 [Goetghebuer 1935: 151]. Type locality: Democratic Republic of the Congo.
Forcipomyia grisescens Goetghebuer, 1935 [Goetghebuer 1935: 154]. Type locality: Democratic Republic of the Congo.
Forcipomyia vicina Goetghebuer, 1935 [Goetghebuer 1935: 161]. Type locality: Democratic Republic of the Congo.
Forcipomyia longiradialis Tokunaga, 1940 [Tokunaga 1940: 66]. Type locality: Japan.
Forcipomyia takagii Tokunaga, 1941 [*Tokunaga 1941*: 90]. Type locality: China.
Lasiohelea wansoni Harant and Baur, 1946 [Harant and Baur 1946: 141], preoccupied by *Forcipomyia wansoni*De Meillon 1939. Type locality: Democratic Republic of the Congo.

##### Materials

- **Type status:** Other material. **Occurrence:** individualCount: 1; sex: male; lifeStage: adult; disposition: personal collection of El-Hawagry; **Taxon:** scientificName: *Forcipomyia fuliginosa* (Meigen, 1818); kingdom: Animalia; phylum: Arthropoda; class: Insecta; order: Diptera; family: Ceratopogonidae; **Location:** continent: Africa; country: Egypt; stateProvince: Giza; locality: Cairo University; **Event:** samplingProtocol: light trap; eventTime: 15 Jan 1996

##### Distribution

AF: Widely distributed in AF, NE, NEO, OR and PA.

Local distribution in Egypt: Lower Nile Valley & Delta: El-Badrasheen, Giza. Upper Nile. Valley: Assiout. Western Desert: El-Bustan.

Dates of collection in Egypt: December & April.

#### Synthyridomyia

Saunders, 1957

4D2BAF1C-B7B7-5E01-A56A-76510C105756

Synthyridomyia ***Synthyridomyia*** Saunders, 1957 [Saunders 1957: 688]. Type species: *Lasiohelea acidicola* Tokunaga, by original designation.

#### Forcipomyia (Synthyridomyia) murina

(Winnertz, 1852)

387ABAA8-C0A9-5DB0-B070-9B6C4FB7918C

Ceratopogon murinus Winnertz, 1852 [Winnertz 1852: 26]. Type locality: Europe.
Helea murina var. abdominalis Santos Abreu, 1918 [Santos Abreu 1918: 265]. Type locality: Canary Islands (Spain).
Apelma aurosparsum Kieffer, 1919 [Kieffer 1919: 65]. Type locality: Hungary.
Forcipomyia sulfurea Kieffer, 1923 [Kieffer 1923a: 664]. Type locality: Algeria.
Forcipomyia hirtipalpis Kieffer, 1924 [Kieffer 1924b: 392]. Type locality: France.
Forcipomyia sate Kieffer, 1925 [*Kieffer 1925c*: 245]. Type locality: Egypt.
Forcipomyia longitarsis Tokunaga, 1940 [*Tokunaga 1940*: 92], preoccupied by *Forcipomyia longitarsis* (Malloch 1915b). Type locality: Taiwan.
Forcipomyia moascari Macfie, 1943 [Macfie 1943: 147]. Type locality: Egypt.
Forcipomyia attonsa Goetghebuer, 1950 [Goetghebuer 1950: 1]. Type locality: Belgium.
Forcipomyia tokunagai Wirth, 1973: 356 [Wirth 1973], preoccupied by *Forcipomyia tokunagai* (Oka and Asahina 1948). New name for *longitarsis*.
Forcipomyia submurina Remm, 1980 [Remm 1980: 115], as *sibmurina*, as subspecies of *murina*. Type locality: Russia.

##### Distribution

Widespread in AF, PA and NE.

Local distribution in Egypt: Coastal Strip: Alexandria (Sidi Gaber). Eastern Desert: Ismailia (Moascar), Suez (Guyot Garden). Lower Nile Valley & Delta: El-Qanatir, Maadi. Upper Nile Valley: Asswan.

Dates of collection in Egypt: August to April.

#### DASYHELEINAE

7CE45B07-888C-52E2-A237-BFDD1DC43DCC

#### Dasyhelea

Kieffer, 1911

58AA145A-DC1E-504D-911C-B5531F660794

https://www.gbif.org/species/1635616

Dasyhelea ***Dasyhelea*** Kieffer, 1911 [Kieffer 1911b: 5]. Type species: *Dasyhelea halophila* Kieffer, by monotypy.
Prokempia Kieffer, 1913 [*Kieffer 1913c*: 163], 179. Type species: *Dasyhelea ornaticornis* Kieffer, by subsequent designation of Wirth (1973: 358).
Pseudoculicoides Malloch, 1915 [Malloch 1915a: 309]. Type species: *Ceratopogon mutabilis* Coquillett, by original designation.
Tetrahelea Kieffer, 1925 [*Kieffer 1925a*: 423]. Type species: *Culicoides insignicornis* Kieffer, by original designation.
Dicryptoscena Enderlein, 1936 [*Enderlein 1936*: 51]. Type species: *Dasyhelea inclusa* Kieffer, by original designation.
Sebessia Remm, 1979 [*Remm 1979*: 55]. Type species: *Dasyhelea flavopyga* Zilahi-Sebess, by original designation.
Borkentimyia Yu, Liu, Liu, Liu, Hao, Yan and Zhao, 2005 [*Yu et al. 2005*: 321]. Type species: *Dasyhelea forsteri* Grogan and Wirth, by original designation.

#### Dasyhelea arenivaga

Macfie, 1943

04873C7B-CC68-5C87-B3F0-D703055D9D7D

Dasyhelea inconspicuosa var. arenivaga Macfie, 1943 [Macfie 1943: 151]. Type locality: Egypt (Ismailia).

##### Distribution

PA: Algeria, Bulgaria, Czech Republic, Egypt, Israel, Poland, Romania, Spain, Switzerland, Ukraine.

Local distribution in Egypt: Eastern Desert: Ismailia (Moascar). Upper Nile Valley: Asswan.

Dates of collection in Egypt: November to March.

#### Dasyhelea arenosa

Kieffer, 1925

576DC570-10F5-5AFD-8FAA-CC2A6F64BCDB

Dasyhelea arenosa Kieffer, 1925 [Kieffer 1925c: 255]. Type locality: Algeria.

##### Distribution

PA: Algeria, Egypt, Spain.

Local distribution in Egypt: Lower Nile Valley & Delta: Pyramids.

Dates of collection in Egypt: April.

#### Dasyhelea begueti

Kieffer, 1922

EE2767A3-1594-5001-85C7-E185735663BA

Dasyhelea begueti Kieffer, 1922 [Kieffer 1922b: 508]. Type locality: Algeria.
Dasyhelea astyla Kieffer, 1922b: 510. Type locality: Algeria.
Dasyhelea hirtipes Kieffer, 1922b: 510 (as variety of *begueti*, preoccupied by *Dasyhelea hirtipes* (Kieffer, 1917). Type locality: Algeria.

##### Distribution

PA: Algeria, Egypt.

Local distribution in Egypt: Coastal Strip: Alexandria (sea shore). Eastern Desert: Suez (Guyot Gardens), Wadis near Helwan. Lower Nile Valley & Delta: Maadi.

Dates of collection in Egypt: April and October.

#### Dasyhelea egypti

Macfie, 1943

1968A345-409C-5721-A429-99BAB506FC8E

Dasyhelea inconspicuosa var. egypti Macfie, 1943 [Macfie 1943: 149]. Type locality: Egypt (Ismailia: Moascar).

##### Distribution

PA: Egypt.

Local distribution in Egypt: Eastern Desert: Ismailia (Moascar), Lower Nile Valley & Delta: Maadi.

Dates of collection in Egypt: February to November.

#### Dasyhelea fusca

Carter, Ingram and Macfie, 1921

D5AAEB6D-7777-5B1E-8662-34C13EAD6AD9

Dasyhelea fusca Carter, Ingram and Macfie, 1921 [Carter et al. 1921b: 204]. Type locality: Ghana.

##### Distribution

AF: Ghana, Madagascar, Mozambique, South Africa. AF: Egypt.

Local distribution in Egypt: Eastern Desert: Ismailia (Moascar).

Dates of collection in Egypt: March and April.

#### Dasyhelea heliophila

Macfie, 1943

910E4CD3-794C-542E-80B6-02F4BB2C97D1

Dasyhelea inconspicuosa var. heliophila Macfie, 1943 [Macfie 1943: 150]. Type locality: Egypt (Ismailia: Nifisha).

##### Distribution

PA: Egypt.

Local distribution in Egypt: Eastern Desert: Ismailia (Nifisha).

Dates of collection in Egypt: July.

#### Dasyhelea ismailiae

Macfie, 1943

E84B2573-8FDD-50EA-B0FC-7ED5AE82E0F5

Dasyhelea ismailiae Macfie, 1943 [Macfie 1943: 152]. Type locality: Egypt.

##### Distribution

AF: South Africa. PA: Egypt.

Local distribution in Egypt: Eastern Desert: Ismailia (Moascar).

Dates of collection in Egypt: February and March.

#### Dasyhelea luteocincta

Kieffer, 1925

1EA05EF7-C8D3-5864-83C5-FE528BC2FDC4

Dasyhelea luteocincta Kieffer, 1925 [*Kieffer 1925c*: 254]. Type locality: Egypt (Maadi).

##### Distribution

PA: Egypt.

Local distribution in Egypt: Lower Nile Valley & Delta: Maadi.

Dates of collection in Egypt: October.

#### Dasyhelea modesta

(Winnertz, 1852)

1044D28C-0173-56C0-9DF2-7AED5AD1666B

Ceratopogon modestus Winnertz, 1852 [Winnertz 1852: 43]. Type locality: Germany.
Ceratopogon aestivus Winnertz, 1852 [Winnertz 1852: 42]. Type locality: Germany.
Dasyhelea longipalpi s Kieffer, 1913 [Kieffer 1913b: 37]. Type locality: Germany.
Dasyhelea inclusa Kieffer, 1918 [*Kieffer 1918b*: 188]. Type locality: Czech Republic.
Dasyhelea strobli Kieffer, 1919 [Kieffer 1919: 63]. Type locality: Spain.
Dasyhelea pratensis Goetghebuer, 1920 [Goetghebuer 1920: 44]. Type locality: Belgium.
Dasyhelea bihamata Kieffer, 1923 [Kieffer 1923a: 667]. Type locality: Algeria.
Dasyhelea moascari Macfie, 1943 [Macfie 1943: 153]. Type locality: Egypt.
Dasyhelea densipilosa Tokunaga, 1963 [Tokunaga 1963: 41]. Type locality: Japan.

##### Distribution

AF: Yemen. PA: Afghanistan, Algeria, Andorra, Austria, Azerbaijan, Belgium, Bulgaria, Canary Is., China, Czech Republic, Egypt, Estonia, France, Georgia, Germany, Great Britain, Hungary, Iran, Japan, Lithuania, The Netherlands, Norway, Poland, Romania, Russia, Spain, Sweden, Switzerland, Ukraine.

Local distribution in Egypt: Eastern Desert: Ismailia (Moascar). Lower Nile Valley & Delta: EI-Qanatir, EI-Mansoura, Marsafa. Western Desert: EI-Bustan (Beheira).

Dates of collection in Egypt: February, April, August and November.

#### Dasyhelea nyasae

Ingram and Macfie, 1925

1BB3D77E-7C7B-5147-8196-FF2DC1304321

Dasyhelea nyasae Ingram and Macfie, 1925 [Ingram and Macfie 1925: 286]. Type locality: Malawi.

##### Distribution

AF: Malawi, Uganda. PA: Egypt.

Local distribution in Egypt: Eastern Desert: Ismailia (Moascar). Western Desert: EI-Bustan (Beheira).

Dates of collection in Egypt: February.

#### CERATOPOGONINAE

34F55A0C-5BE7-5A56-B575-FF830837DE09

#### CULICOIDINI

29D023E0-662B-5252-8BD8-0517A3D7D4F5

#### Culicoides

Latreille, 1809

3341A8B7-1FC6-5C9A-B16D-E3F23C3F6203

https://www.gbif.org/species/9951338

Culicoides ***Culicoides*** Latreille, 1809 [Latreille 1809: 251]. Type species: *Culicoides punctatus* Latreille, by monotypy.
Padrosia Rafinesque, 1815 [*Rafinesque 1815*: 130], unnecessary new name for *Culicoides* Latreille. Type species: *Culicoides punctatus* Latreille, automatic.
Oecacta Poey, 1853 [*Poey 1853*: 238]. Type species: *Oecacta furens* Poey, by monotypy.
Psychophaena Philippi, 1865 [*Philippi 1865*: 628]. Type species: *Psychophaena pictipennis* Philippi (= *Culicoides venezuelensis* Ortiz and Mirsa), by monotypy.
Haematomyidium Goeldi, 1905 [*Goeldi 1905*: 137]. Type species: *Haematomyidium paraense* Goeldi, by original designation.
Cotocripus Brèthes, 1912 [*Brèthes 1912*, 1912: 451]. Type species: *Cotocripus caridei* Brèthes, by monotypy.
Oxyhelea Kieffer, 1921 [*Kieffer 1921a*: 14]. Type species: *Culicoides dentatus* Kieffer, by monotypy.
Diplosella Kieffer, 1921 [*Kieffer 1921b*: 113]. Type species: *Culicoides sergenti* Kieffer, by monotypy.
Haemophoructus Macfie, 1925 [*Macfie 1925*: 349]. Type species: *Haemophoructus maculipennis* Macfie, by monotypy.
Prosapelma Kieffer, 1925 [*Kieffer 1925a*: 417]. Type species: *Prosapelma cinerea* Kieffer, by original designation.
Synhelea Kieffer, 1925 [*Kieffer 1925a*: 423]. Type species: *Culicoides tropicalis* Kieffer, by subsequent designation of Wirth et al. (1980).
Hoffmania Fox, 1948 [*Fox 1948*: 21]. Type species: *Culicoides inamollae* Fox and Hoffman (= *Culicoides insignis* Lutz), by original designation.
Beltranmyia Vargas, 1953 [*Vargas 1953*: 34]. Type species: *Culicoides crepuscularis* Malloch, by original designation.
Selfia Khalaf, 1954 [*Khalaf 1954*: 38]. Type species: *Culicoides hieroglyphicus* Malloch, by original designation.
Monoculicoides Khalaf, 1954 [*Khalaf 1954*: 39]. Type species: *Ceratopogon nubeculosus* Meigen, by original designation.
Macfiella Fox, 1955 [*Fox 1955*: 217]. Type species: *Ceratopogon phlebotomus* Williston, by original designation.
Avaritia Fox, 1955 [*Fox 1955*: 218]. Type species: *Ceratopogon obsoletus* Meigen, by original designation.
Trithecoides Wirth and Hubert, 1959 [*Wirth and Hubert 1959*: 2]. Type species: *Culicoides flaviscutatus* Wirth and Hubert, by original designation.
Anilomyia Vargas, 1960 [*Vargas 1960*: 37]. Type species: *Culicoides covagarciai* Ortiz, by original designation.
Drymodesmyia Vargas, 1960 [*Vargas 1960*: 40]. Type species: *Culicoides copiosus* Root and Hoffman, by original designation.
Diphaomyia Vargas, 1960 [*Vargas 1960*: 40]. Type species: *Culicoides baueri* Hoffman, by original designation.
Glaphiromyia Vargas, 1960 [*Vargas 1960*: 41]. Type species: *Culicoides scopus* Root and Hoffman, by original designation.
Mataemyia Vargas, 1960 [*Vargas 1960*: 43]. Type species: *Culicoides mojingaensis* Wirth and Blanton, by original designation.
Meijerehelea Wirth and Hubert, 1961 [*Wirth and Hubert 1961*: 23]. Type species: *Ceratopogon guttifer* de Meijere, by original designation.
Pontoculicoides Remm, 1968 [Remm and Zhogolev 1968: 840]. Type species: *Culicoides tauricus* Gutsevich, by original designation.
Callotia Vargas and Kremer, 1972 [Vargas and Kremer 1972: 242]. Type species: *Culicoides saevus* Kieffer, by original designation.
Wirthomyia Vargas, 1973 [*Vargas 1973*: 112]. Type species: *Culicoides segnis* Campbell and Pelham Clinton, by original designation.
Sensiculicoides Shevchenko, 1977 [*Shevchenko 1977*: 133]. Type species: *Ceratopogon pictipennis* Staeger, by original designation.
Remmia Glukhova, 1977 [*Glukhova 1977*: 116]. Type species: *Ceratopogon schultzei* Enderlein, by original designation.
Silvaticulicoides Glukhova, 1977 [*Glukhova 1977*: 117]. Type species: *Ceratopogon fascipennis* Staeger, by original designation.
Neoculicoides Boorman and Lane, 1979 [*Boorman and Lane 1979*: 327], preoccupied by *Neoculicoides* Pierce, 1966. Type species: *Neoculicoides taylori* Boorman and Lane, by original designation.
Nullicella Lee, 1982 [*Lee 1982*: 165]. Type species: *Culicoides lasaensis* Lee, (by original designation).
Sinocoides Chu, 1983 [*Chu 1983*: 26]. Type species: *Culicoides hamiensis* Chu, Qian and Ma, by original designation.
Jilinocoides Chu, 1983 [*Chu 1983*: 28]. Type species: *Culicoides dunhuaensis* Chu, by original designation.
Amossovia Glukhova, 1989 [*Glukhova 1989*: 226]. Type species: *Culicoides dendrophilus* Amosova, by original designation.
Silvicola Mirzaeva and Isaev, 1990 [*Mirzaeva and Isaev 1990*: 98]. Type species: *Culicoides grisescens* Edwards, by original designation.
Tokunagahelea Dyce and Meiswinkel, 1995 [*Dyce and Meiswinkel 1995*: 131]. Type species: *Culicoides mikros* Dyce and Meiswinkel, by original designation.
Fastus Liu, 2005 [Yu et al. 2005: 1196]. Type species: *Culicoides alpigenus* Yu and Liu, by original designation.
Marksomyia Bellis and Dyce, 2011 [*Bellis and Dyce 2011*: 36]. Type species: *Culicoides marksi* Lee and Reye, by original designation.
Devalquia Choufani and Nel, 2013 [Choufani et al. 2013: 75]. Type species: *Devalquia brisaci* Choufani & Nel, 2013, by original designation.

#### Culicoides azerbajdzhanicus

Dzhafarov, 1962

C6AFA876-342C-5A6F-B808-1601C7B4B299

Culicoides azerbajdzhanicus Dzhafarov, 1962 [Dzhafarov 1962: 211]. Type locality: Azerbaijan.

##### Distribution

AF: Senegal. PA: Algeria, Armenia, Azerbaijan, Cyprus, Egypt, Iran, Israel, Morocco, Turkmenistan, Uzbekistan.

Local distribution in Egypt: Sinai: El-Tour, Marsa Abu Zabad, Wadi Ba'aba'a (S. Sinai).

Dates of collection in Egypt: Unknown.

#### Culicoides bedfordi

Ingram and Macfie, 1923

19470182-FFF3-5FE6-8106-D7C10895A7B8

Culicoides bedfordi Ingram and Macfie, 1923 [Ingram and Macfie 1923: 57]. Type locality: South Africa.

##### Distribution

AF: Cameroon, Kenya, South Africa, Sudan, Tanzania, Zimbabwe. PA: Egypt.

Local distribution in Egypt: Lower Nile Valley & Delta: EI-Hamra, EI-Qanatir. Upper Nile Valley: Luxor. Western Desert: Baharriya Oasis (EI-Hara), Wadi El-Natroun.

Dates of collection in Egypt: August to December.

#### Culicoides calloti

Kremer, Delécolle, Bailly-Choumara and Chaker, 1979

88EA3CA0-169F-56E1-8AD6-DDA7B7822013

Culicoides calloti Kremer, Delécolle and Bailly-Choumara and Chaker, 1979 [*Kremer et al. 1979*: 195]. Type locality: Morocco (Tarhjicht).

##### Distribution

PA: Egypt, Israel, Morocco.

Local distribution in Egypt: Sinai: El-Tour (Wadi Dissa), Hammam Moussa.

Dates of collection in Egypt: Unknown.

#### Culicoides cataneii

Clastrier, 1957

259EE0A3-0EB7-52B6-98EF-7232FC60D542

Culicoides cataneii Clastrier, 1957 [Clastrier 1957: 438]. Type locality: Algeria

##### Distribution

PA: Algeria, Bulgaria, Cyprus, Egypt, France, Iraq, Iran, Israel, Italy, former Yugoslavia, Morocco, Russia, Turkmenistan, Ukraine.

Local distribution in Egypt: Sinai: El-Arish.

Dates of collection in Egypt: Unknown.

#### Culicoides catharinae

Kremer, Delécolle and Braverman, 1991

0E877CEA-3FBE-5188-BE50-033AC3E575D3

Culicoides catharinae Kremer, Delécolle and Braverman, 1991 [Kremer et al. 1991: 151]. Type locality: Egypt.

##### Distribution

PA: Egypt.

Local distribution in Egypt: Sinai: St. Catherine (S. Sinai).

Dates of collection in Egypt: June.

#### Culicoides circumscriptus

Kieffer, 1918

70C472BA-793B-5832-B5D5-73F341B733E9

Culicoides circumscriptus Kieffer, 1918 [*Kieffer 1918a*: 49]. Type locality: Tunisia.
Culicoides nadayanus Kieffer, 1918 [*Kieffer 1918a*: 95]. Type locality: Turkey.
Culicoides edwardsi Goetghebuer, 1921 [Goetghebuer 1921: 177]. Type locality: Belgium.
Culicoides algarum Kieffer, 1924 [Kieffer 1924a: 18]. Type locality: Germany.
Culicoides salicola Kieffer, 1924 [Kieffer 1924b: 405]. Type locality: Norway.
Culicoides salicola var. pictidorsum Kieffer, 1924 [Kieffer 1924b: 406]. Type locality: Norway.
Culicoides albonotatus Vimmer, 1932 [Vimmer 1932: 133], preoccupied by *Culicoides albonotatus* Kieffer 1918. Type locality: Israel.
Culicoides albosignatus Vimmer, 1932 [Vimmer 1932: 135]. Type locality: Israel.
Culicoides polymaculatus Vimmer, 1932 [Vimmer 1932: 135]. Type locality: Israel.
Culicoides pulcher Zilahi-Sebess, 1934 [Zilahi-Sebess 1934: 155], misspelled as *pulscher*. Type locality: Bulgaria.
Culicoides kirovabadicus Dzhafarov, 1964 [Dzhafarov 1964: 228]. Type locality: Azerbaijan.
Culicoides circumscriptus matsuensis Lien, Weng and Lin, 1996 [Lien et al. 1996: 20]. Type locality: Taiwan.
Culicoides circumscriptus meridionalis Xue, Liu and Yu, 2003 [Xue et al. 2003: 112]. Type locality: China.

##### Materials

- **Type status:** Other material. **Occurrence:** individualCount: 1; sex: female; lifeStage: adult; disposition: personal collection of El-Hawagry; **Taxon:** scientificName: *Culicoides circumscriptus* Kieffer, 1918; kingdom: Animalia; phylum: Arthropoda; class: Insecta; order: Diptera; family: Ceratopogonida; **Location:** continent: Africa; country: Egypt; stateProvince: Giza; locality: El-Hager; **Event:** samplingProtocol: light trap; eventDate: 15 Aug 1996

##### Distribution

OR & PA: Widespread in Asia and Europe, Egypt, Tunisia.

Local distribution in Egypt: Coastal Strip: Abis. Eastern Desert: Ismailia (Moascar). Fayoum: EI-Siliyin, Lower Nile Valley & Delta: Abbassiya (Cairo), Abu-Rawash, Aga, El-Hager (Mansheyet El-Qanater), EI-Qanatir, Mit Yaish (Mit Ghamr), Nawasa El-Gheit. Upper Nile Valley: Assiout (Biblaw), Minya. Western Desert: Abis, El-Bustan.

Dates of collection in Egypt: March to November.

#### Culicoides distinctipennis

Austen, 1912

11C04EFE-2A1E-5B85-A128-8C46FE0B3774

Culicoides distinctipennis Austen, 1912 [Austen 1912: 101]. Type locality: Nigeria.
Forcipomyia multiguttata Goetghebuer, 1935 [Goetghebuer 1935: 156]. Type locality: Democratic Republic of the Congo.

##### Materials

- **Type status:** Other material. **Occurrence:** individualCount: 1; sex: female; lifeStage: adult; disposition: personal collection of El-Hawagry; **Taxon:** scientificName: *Culicoides distinctipennis* Austen, 1912; kingdom: Animalia; phylum: Arthropoda; class: Insecta; order: Diptera; family: Ceratopogonida; **Location:** continent: Africa; country: Egypt; stateProvince: El-Dakahlyia; locality: El-Mansoura; **Event:** samplingProtocol: light trap; eventDate: 17 Nov 199
- **Type status:** Other material. **Occurrence:** individualCount: 1; sex: male; lifeStage: adult; disposition: personal collection of El-Hawagry; **Taxon:** scientificName: *Culicoides distinctipennis* Austen, 1912; kingdom: Animalia; phylum: Arthropoda; class: Insecta; order: Diptera; family: Ceratopogonida; **Location:** continent: Africa; country: Egypt; stateProvince: El-Dakahlyia; locality: El-Mansoura; **Event:** samplingProtocol: light trap; eventDate: 17 Nov 199

##### Distribution

AF: Widespread. PA: Egypt.

Local distribution in Egypt: Eastern Desert: Ismailia. Fayoum: EI-Siliyin. Lower Nile Valley & Delta: Abbassiya, Abu-Rawash, Ashmoun, Ayiat, Bulaq El-Dakrour, Cairo, El-Khanka, El-Mansoura, EI-Qanatir, Etay El-Baroud, Kafr Hamza, Kafr Tarkhan, Maadi, Mit Yaish (Mit Ghamr), Shatanuf. Upper Nile Valley: Assiout. Western Desert: El-Bustan, Itay El-Baroud.

Dates of collection in Egypt: May to December.

#### Culicoides firuzae

Dzhafarov, 1958

AD3E816E-2673-59AB-A802-94764EA0D77D

Culicoides firuzae Dzhafarov, 1958 [*Dzhafarov 1958*: 245]. Type locality: Azerbaijan (Araks Valley).

##### Distribution

PA: Armenia, Azerbaijan Egypt, Georgia, Iran, Israel, Kazakhstan, Mongolia, Russia, Tajikistan, Turkmenistan, Uzbekistan.

Local distribution in Egypt: Sinai: Wadi Ba’aba’a.

Dates of collection in Egypt: Unknown.

#### Culicoides hanae

Braverman, Delécolle and Kremer, 1983

A8C5D5F2-EEAC-52A2-A608-233B61F86FC5

Culicoides hanae Braverman, Delécole and Krémer, 1983 [Braverman et al. 1983: 678]. Type locality: Egypt (Sinai: Qadesh Barnea).

##### Distribution

PA: Egypt, Israel.

Local distribution in Egypt: Sinai: Qadesh Barnea (El-Arish).

Dates of collection in Egypt: November.

#### Culicoides imicola

Kieffer, 1913

E4F8C17A-CB94-53B7-B19C-27063ADF220A

Culicoides imicola Kieffer, 1913 [Kieffer 1913d: 11]. Type locality: Kenya.
Ceratopogon pallidipennis Carter, Ingram and Macfie, 1920 [Carter et al. 1920: 265]. Type locality: Ghana.
Culicoides iraqensis Khalaf, 1957 [*Khalaf 1957*: 343]. Type locality: Iraq.
Culicoides minutus Sen and Das Gupta, 1959 [Sen and Das Gupta 1959: 622]. Type locality: India.
Culicoides pseudoturgidus Das Gupta, 1962 [Das Gupta 1962: 537]. Type locality: India.

##### Distribution

AF: Widespread. OR: India, Laos, Sri Lanka, Thailand, Vietnam. PA: Algeria, Cyprus, France, the Near and Middle East, Morocco, Portugal, Spain.

Local distribution in Egypt: Eastern Desert: Ismailia (Moascar), Suez. Lower Nile Valley & Delta: Giza, Bulaq El-Dakrour.

Dates of collection in Egypt: February to May.

#### Culicoides jumineri

Callot and Kremer, 1969

466B11B2-9432-58CA-97DE-04500C935BF9

Culicoides jumineri Callot and Krémer, 1969 [*Callot and Kremer 1969*: 1112]. Type locality: Tunisia.

##### Distribution

PA: Algeria, Corsica, Egypt, Israel, Morocco, Tunisia, Portugal, Spain, Turkey.

Local distribution in Egypt: Sinai: Ga’al.

Dates of collection in Egypt: Unknown.

#### Culicoides kingi

Austen, 1912

655DB69B-B404-5662-B3E0-A9C0229E8EA1

Culicoides kingi Austen, 1912 [Austen 1912: 104]. Type locality: Sudan (Khor Arbaat, Red sea Hills).
Culicoides nilotes Kieffer, 1925 [*Kieffer 1925c*: 257]. Type locality: Egypt.

##### Distribution

AF: Sudan. PA: Egypt.

Local distribution in Egypt: Eastern Desert: Ismailia (Moascar). Lower Nile Valley & Delta: Maadi. Sinai: Bir El-Abd.

Dates of collection in Egypt: February to April and August to November.

#### Culicoides langeroni

Kieffer, 1921

14703D02-323D-56E4-9262-5A8385C2519D

Culicoides langeroni Kieffer, 1921 [Kieffer 1921c: 262]. Type locality: Tunisia.

##### Distribution

PA: Egypt, Iran, Israel, Tunisia.

Local distribution in Egypt: Sinai: Bir El-Abd, El-Tour, Hammam Moussa.

Dates of collection in Egypt: July.

#### Culicoides leucostictus

Kieffer, 1911

67AB3436-6CD8-5001-BCA6-6A5652C75AB7

Culicoides leucostictus Kieffer, 1911 [Kieffer 1911b: 340]. Type locality: Seychelles.
Culicoides praetermissus Carter, Ingram and Macfie, 1920 [Carter et al. 1920: 240]. Type locality: Ghana.
Culicoides distinctipennis var. egypti Macfie, 1924 [Macfie 1924: 66]. Type locality: Egypt (Nile at Assiout).
Culicoides pharao Kieffer, 1925 [*Kieffer 1925c*: 259]. Type locality: Egypt (Maadi).

##### Distribution

AF: Widespread. PA: Cyprus, Egypt, Israel.

Local distribution in Egypt: Eastern Desert: Ismailia (Moascar). Lower Nile Valley & Delta: Maadi. Upper Nile Valley: Assiout, Minya. Western Desert: Wadi El-Natroun.

Dates of collection in Egypt: October to April.

#### Culicoides marcleti

Callot, Kremer and Basset, 1968

BD87F2CB-0362-5B5B-9303-BB59E292BC89

Culicoides marcleti Callot, Krémer and Basset, 1968 [Callot et al. 1968: 271]. Type locality: Algeria.

##### Distribution

PA: Algeria, Egypt, Morocco, Spain, Tunisia.

Local distribution in Egypt: Sinai: Khirba.

Dates of collection in Egypt: Unknown.

#### Culicoides maritimus

Kieffer, 1924

D501C17D-22FB-571A-92FC-80EB6F50A8E6

Culicoides maritimus Kieffer, 1924 [Kieffer 1924a: 16]. Type locality: Germany (Schleswig-Holstein: Holstein).
Culicoides submaritimus Dzhafarov, 1962 [Dzhafarov 1962: 206]. Type locality: Azerbaijan.

##### Distribution

PA: Europe (Widespread), Middle East and North Africa.

Local distribution in Egypt: Sinai: Bir El-Abd, Salmana, Ga’al, Khirba.

Dates of collection in Egypt: Unknown.

#### Culicoides mesghalii

Navai, 1973

1CF4A07F-CAD3-5895-A758-1DE2E83E9C0F

Culicoides mesghalii Naval, 1973 [*Navai 1973*: 196]. Type locality: Iran.

##### Distribution

PA: Egypt, Iran, Israel.

Local distribution in Egypt: Sinai: Mersa Abu Zabad.

Dates of collection in Egypt: Unknown.

#### Culicoides navaiae

Lane, 1983

C766E1C5-4AFE-5665-8EA0-E45E9646CF72

Culicoides navaiae Lane, 1983 [Lane 1983: 534]. Type locality: Saudi Arabia.

##### Distribution

PA: Egypt, Saudi Arabia.

Local distribution in Egypt: Sinai: Khirba.

Dates of collection in Egypt: April.

#### Culicoides neavei

Austen, 1912

24E90EFA-06D5-58E5-9535-765E7E39B558

Culicoides neavei Austen, 1912 [Austen 1912: 102]. Type locality: Uganda.

##### Ecological interactions

###### Native status

Doubtful record.

##### Distribution

AF: Angola, Cameroon, Congo, Ethiopia, Gambia, Ghana, Guinea, Kenya, Madagascar, Mali, Nigeria, Senegal, South Africa, Sudan, Tanzania, Uganda, Upper Volta, Zaire, Zimbabwe. PA: ?Egypt.

Local distribution in Egypt: ?Eastern Desert: ?Ismailia.

##### Notes

?- This Afrotropical species was listed as recorded from Egypt by Morsy et al. (1989), but no specimens have been found to ensure this record. It must be considered very doubtful as an Egyptian species.

#### Culicoides newsteadi

Austen, 1921

0A2554B8-9EBD-59DF-9131-0A245D33CC41

Culicoides newsteadi Austen, 1921 [*Austen 1921*: 113]. Type locality: Israel (Jerisheh).
Culicoides biclavatus Kieffer, 1924 [Kieffer 1924a: 14]. Type locality: Germany.
Culicoides halophilus Kieffer, 1924 [Kieffer 1924b: 404]. Type locality: Norway.
Culicoides pulicaris var. edwardsi Goetghebuer, 1933 [*Goetghebuer 1933c*: 46], preoccupied by *Culicoides edwardsi* Goetghebuer, 1921. Type locality: Europe.
Culicoides pulicaris var. edwardsianus Goetghebuer, 1933 [Goetghebuer 1933a: 367]. Type locality: Great Britain.

##### Distribution

PA: Algeria, Egypt, Europe (distributed through much of Europe), Iran, Iraq, Israel, Morocco.

Local distribution in Egypt: Sinai: Sheikh Zowaiid (N. Sinai). Western Desert: El-Bustan.

Dates of collection in Egypt: February and March.

#### Culicoides pilosipennis

Kieffer, 1925

4124DD5F-6660-5810-9557-30FAE39748D4

Culicoides pilosipennis Kieffer, 1925 [*Kieffer 1925c*: 257]. Type locality: Egypt.

##### Distribution

PA: Egypt.

Local distribution in Egypt: Eastern Desert: Suez (Guyot Garden).

Dates of collection in Egypt: April.

#### Culicoides pulicaris

(Linnaeus, 1758)

C1CD0705-BCDF-5E0D-8687-4E6BE7DF6167

Culex pulicaris Linnaeus, 1758 [Linnaeus 1758: 603]. Type locality: Europe.
Culicoides setosinervis Kieffer, 1913 [Kieffer 1913a: 8]. Type locality: Germany.
Culicoides pullatus Kieffer, 1915 [Kieffer 1915: 474]. Type locality: Germany.
Culicoides stephensi Carter, 1916 [Carter 1916: 135]. Type locality: Egypt.
Culicoides cinerellus Kieffer, 1919 [Kieffer 1919: 40]. Type locality: Ukraine.
Culicoides quinquepunctatus Goetghebuer, 1921 [Goetghebuer 1921: 177]. Type locality: Belgium.
Culicoides flaviplumus Kieffer, 1924 [Kieffer 1924a: 19]. Type locality: Germany.
Culicoides sawamotoi Kono and Takahasi, 1940 [*Kono and Takahasi 1940*: 75.] Type locality: Russia.

##### Distribution

PA: Widespread.

Local distribution in Egypt: Eastern Desert: Ismailia (Moascar). Sinai: Bir El-Abd.

Dates of collection in Egypt: March to July.

#### Culicoides puncticollis

(Becker, 1903)

2420AE0B-4C1A-5268-BA4D-ACD5982DD5B8

Ceratopogon puncticollis Becker, 1903 [Becker 1903: 75]. Type locality: Egypt.
Ceratopogon pulicaris form algecirensis Strobl, 1900 [*Strobl 1900*: 170]. Type locality: Spain. Name suppressed by ICZN Opinion 1643.
Culicoides impressus Kieffer, 1918 [*Kieffer 1918a*: 47]. Type locality: Tunisia.
Culicoides distigma Kieffer, 1922 [Kieffer 1922b: 502]. Type locality: Algeria.
Culicoides donatieni Kieffer, 1922 [Kieffer 1922b: 504]. Type locality: Algeria.
Culicoides sciniphes Kieffer, 1925 [*Kieffer 1925c*: 261]. Type locality: Egypt.
Culicoides bipunctatus Vimmer, 1932 [Vimmer 1932: 133]. Type locality: Israel.
Culicoides tripunctatus Vimmer, 1932 [Vimmer 1932: 137]. Type locality: Israel.
Culicoides wenigi Vimmer, 1932 [Vimmer 1932: 138]. Type locality: Israel.
Culicoides flavitarsis Vimmer, 1932 [Vimmer 1932: 137]. Type locality: Israel.
Culicoides griseovittatus Vimmer, 1932 [Vimmer 1932: 133]. Type locality: Israel.
Culicoides luteosignatus Vimmer, 1932 [Vimmer 1932: 140]. Type locality: Israel.
Culicoides vavrai Vimmer, 1932 [Vimmer 1932: 140]. Type locality: Israel.

##### Materials

- **Type status:** Other material. **Occurrence:** individualCount: 1; sex: male; disposition: personal collection of El-Hawagry; **Taxon:** scientificName: *Culicoides puncticollis* (Becker, 1903); kingdom: Animalia; phylum: Arthropoda; class: Insecta; order: Diptera; family: Ceratopogonidae; **Location:** continent: Africa; country: Egypt; stateProvince: El-Dakahlyia; locality: Sallent, El-Mansoura; **Event:** samplingProtocol: light trap; eventDate: 17 Nov 1997

##### Distribution

PA: Balearic Is., Belgium, British Is., Corsica, Cyprus, Dodecanese Is., France, Hungary, Italy, Middle East, North Africa, Poland, Portugal, Russia, Slovakia, Spain, Turkey, Ukraine.

Local distribution in Egypt: Coastal Strip: Alexandria, Burg El-Arab, Burg Rashid. Eastern Desert: Ismailia (Fayed, Nifisha & Moascar), Suez. Fayoum: Sinnuris. Lower Nile Valley & Delta: Abbassiya, Abu-Rawash, Aga, Bulaq El-Dakrour, Cairo, Desouk, El-Basatin, EI-Mansoura, Giza, Maadi, Mit Yaish (Mit Ghamr). Upper Nile Valley: Dairout, Minya. Western Desert: Abis, Itay El-Baroud.

Dates of collection in Egypt: March to November.

#### Culicoides riethi

Kieffer, 1914

802A84DD-3E11-546A-8C1B-7216D07E8769

Culicoides riethi Kieffer, 1914 [Kieffer 1914a: 237]. Type locality: Germany.
Culicoides cordatus Kieffer, 1921 [*Kieffer 1921b*: 114 (1921d: 275)]. Type locality: Latvia.
Culicoides crassiforceps Kieffer, 1924 [Kieffer 1924a: 15]. Type locality: Germany.
Culicoides gigas Root and Hoffman, 1937 [Root and Hoffman 1937: 172]. Type locality: Canada (Saskatchewan).

##### Distribution

NE: from Alaska to Manitoba, south to British Columbia, Wyoming and Nebraska. PA: China, Egypt, Europe (widespread), Iran, Japan, Mongolia, Morocco.

Local distribution in Egypt: Coastal Strip: Roseta. Fayoum: El-Siliyin Lower Nile valley & Delta: Abu-Rawash. Upper Nile Valley: Asswan (Botanical Garden, High Dam), Minya. Western Desert: Wadi El-Natroun.

Dates of collection in Egypt: throughout the year.

#### Culicoides sahariensis

Kieffer, 1923

981F1013-F2CB-5111-B7DE-357B81AEEE63

Culicoides sahariensis Kieffer, 1923 [Kieffer 1923a: 678]. Type locality: Algeria.
Culicoides similis baghdadensis Khalaf, 1957 [*Khalaf 1957*: 341]. Type locality: Iraq.
Culicoides coluzzii Callot, Kremer and Bailly-Choumara, 1970 [*Callot et al. 1970*: 710]. Type locality: Tunisia.

##### Distribution

PA: Algeria, Cyprus, Italy, Portuguese, Sicily, Spain, Middle East, Turkey.

Local distribution in Egypt: Sinai: Bir El-Abd, Bir Lahfan.

Dates of collection in Egypt: Unknown.

#### Culicoides schultzei

(Enderlein, 1908)

6E6A5622-D429-5397-BB58-305A6552B288

Ceratopogon schultzei Enderlein, 1908 [Enderlein 1908: 459]. Type locality: Namibia.
Culicoides irroratus Goetghebuer, 1948 [Goetghebuer 1948: 12]. Type locality: Democratic Republic of the Congo.

##### Materials

- **Type status:** Other material. **Occurrence:** individualCount: 1; sex: male; lifeStage: adult; **Taxon:** scientificName: *Culicoides schultzei* (Enderlein, 1908); kingdom: Animalia; phylum: Arthropoda; class: Insecta; order: Diptera; family: Ceratopogonidae; **Location:** continent: Africa; country: Egypt; stateProvince: El-Behaira; locality: Wadi El-Natrou; **Event:** samplingProtocol: light trap; eventDate: 3 May 1993
- **Type status:** Other material. **Occurrence:** individualCount: 1; sex: fmale; lifeStage: adult; **Taxon:** scientificName: *Culicoides schultzei* (Enderlein, 1908); kingdom: Animalia; phylum: Arthropoda; class: Insecta; order: Diptera; family: Ceratopogonidae; **Location:** continent: Africa; country: Egypt; stateProvince: El-Dakahlyia; locality: Sellent, El-Mansoura; **Event:** samplingProtocol: light trap; eventDate: 17 Nov 199

##### Distribution

AF: Widespread. AU: New Ginea. OR: Indonesia. PA: China, Cyprus, Japan, Korea, Middle East, North Africa, Tajikistan, Turkmenistan, Uzbekistan.

Local distribution in Egypt: widely distributed.

Dates of collection in Egypt: throughout the year.

#### Culicoides sejfadinei

Dzhafarov, 1958

81A9AF8A-5346-531A-AE9D-A5957625927A

Culicoides sejfadinei Dzhafarov, 1958 [Dzhafarov 1958: 247]. Type locality: Azerbaijan.
Culicoides flavidus Dzhafarov, 1959 [Dzhafarov 1959: 470]. Type locality: Azerbaijan

##### Distribution

PA: Algeria, Armenia, Azerbaijan, Bosnia and Herzegovina, China, France, Greece, Italy, Kyrgyzstan, Middle East, Morocco, Tajikistan, Turkey, Turkmenistan, Uzbekistan, former Yugoslavia.

Local distribution in Egypt: Sinai: Wadi Sa’al.

Dates of collection in Egypt: Unknown.

#### Culicoides sergenti

(Kieffer, 1921)

8B34BA36-7EC3-5578-A2C5-E19BD4E6D87D

Diplosella sergenti Kieffer, 1921 [*Kieffer 1921b*: 113]. Type locality: Algeria (El-Outaya).
Culicoides citrinellus Kieffer, 1923 [Kieffer 1923a: 674]. Type locality: Algeria.
Culicoides mosulensis Khalaf, 1957 [*Khalaf 1957*: 339]. Type locality: Iraq.
Culicoides turkmenicus Gutsevich, 1959 [*Gutsevich 1959*: 678]. Type locality: Turkmenistan.

##### Distribution

PA: Algeria, Cyprus, Egypt, France, Iran, Iraq, Israel, Russia, Tajikistan, Turkmenistan, Uzbekistan.

Local distribution in Egypt: Sinai: El-Tour, Wadi Ba’aba’a.

Dates of collection in Egypt: Unknown.

#### Culicoides similis

Carter, Ingram and Macfie, 1920

94E2F09A-79DF-58DE-8EE3-61524856C5A3

Culicoides similis Carter, Ingram and Macfie, 1920 [Carter et al. 1920: 255]. Type locality: Ghana.

##### Distribution

AF: Widespread. OR: India, Laos, Malaysia, Thailand. PA: Middle East, Morocco.

Local distribution in Egypt: Eastern Desert: Ismailia (Moascar). Lower Nile Valley & Delta: Kafr El-Zaiyat, Shatanouf.

Dates of collection in Egypt: February to July.

#### Culicoides stigma

(Meigen, 1818)

F3126526-AC95-5F8D-A828-60C1B464E5D4

Ceratopogon stigma Meigen, 1818 [Meigen 1818: 73]. Type locality: Europe.
Culicoides kiefferi Goetghebuer, 1910 [Goetghebuer 1910: 96]. Type locality: Belgium.
Culicoides cordiformitarsis Carter, 1916 [Carter 1916: 134]. Type locality: Egypt.
Culicoides unimaculatus Goetghebuer, 1920 [Goetghebuer 1920: 57]. Unnecessary new name for *kiefferi* Goetghebuer.
Culicoides stigmoides Callot, Kremer and Deduit, 1962 [*Callot et al. 1962*: 166]. Type locality: France.

##### Distribution

NE: Canada. PA: Egypt, Europe (widespread).

Local distribution in Egypt: Unknown.

Dates of collection in Egypt: Unknown.

##### Notes

This species was listed as recorded from Egypt by Steyskal & El-Bialy (1967), but no other published records or specimens have been found.

#### Culicoides vitreipennis

Austen, 1921

E45B2F94-0F75-54C0-AD70-B9FDA7DD8DFD

Culicoides vitreipennis Austen, 1921 [*Austen 1921*: 108]. Type locality: Israel (Jerisheh).

##### Distribution

PA: Egypt, Israel.

Local distribution in Egypt: Eastern Desert: Ismailia (Moascar). Sinai: Bir El-Abd.

Dates of collection in Egypt: April to July.

#### CERATOPOGONINI

38B076CC-F367-592B-9AA3-E5B71F593ADE

#### Alluaudomyia

Kieffer, 1913

870ED8DF-5A26-5135-B199-5767BD1D7759

https://www.gbif.org/species/1632585

Alluaudomyia ***Alluaudomyia*** Kieffer, 1913 [Kieffer 1913d: 12]. Type species: *Alluaudomyia imparunguis* Kieffer, by monotypy.
Neoceratopogon Malloch, 1915 [*Malloch 1915b*: 310]. Type species: *Ceratopogon bellus* Coquillett, by original designation.
Prionognathus Carter, Ingram and Macfie, 1921 [*Carter et al. 1921a*: 309]. Type species: *Prionognathus marmoratus* Carter, Ingram and Macfie, by original designation.
Thysanognathus Ingram and Macfie, 1922 [*Ingram and Macfie 1922*: 244]. New name for *Prionognathus* Carter, Ingram and Macfie. Type species: *Prionognathus marmoratus* Carter, Ingram and Macfie, automatic.
Isoecacta Garrett, 1925 [*Garrett 1925*: 9]. Type species: *Isoecacta poeyi* Garrett (= *Ceratopogon bellus* Coquillett), by original designation.

#### Alluaudomyia melanosticta

(Ingram and Macfie, 1922)

6A0B3C90-B607-517D-9445-C36CC2A1E40F

Thysanognathus melanosticta Ingram and Macfie, 1922 [*Ingram and Macfie 1922*: 248]. Type locality: Ghana.
Thysanognathus nilogenes Kieffer, 1925 [*Kieffer 1925c*: 262]. Type locality: Egypt.

##### Distribution

AF: Widespread. PA: Egypt.

Local distribution in Egypt: Eastern Desert: Ismailia. Lower Nile Valley & Delta: Ayiat, Maadi. Upper Nile Valley: Beni Hassan.

Dates of collection in Egypt: October to March.

#### Serromyia

Meigen, 1818

E2C538A0-3DD0-5E06-9A3E-C10473053D13

https://www.gbif.org/species/1633463

Serromyia ***Serromyia*** Meigen, 1818 [Meigen 1818: 83]. Type species: *Ceratopogon femoratus* Meigen, by monotypy.
Prionomyia Stephens, 1829 [*Stephens 1829b*: 237]. Type species: *Ceratopogon femoratus* Meigen, by subsequent designation of Westwood (1840: 126).
Atmobia Bigot, 1857 [*Bigot 1857*: 519]. Type species: *Ceratopogon femoratus* Meigen, by subsequent designation of Evenhuis and Pont (2004).
Ceratolophus Kieffer, 1899 [*Kieffer 1899*: 69], preoccupied by Barboza de Bocage, 1873. Type species: *Ceratopogon femoratus* Meigen, by original designation.
Johannseniella Williston, 1907 [Williston 1907: 1]. New name for *Ceratolophus* Kieffer. Type species: *Ceratopogon femoratus* Meigen, automatic.
Ceratolophana Strand, 1928 [*Strand 1928*: 48]. New name for *Ceratolophus* Kieffer. Type species: *Ceratopogon femoratus* Meigen, automatic.

#### Serromyia mangrovi

Delécolle and Braverman, 1987

9FFDE3DC-0939-55C1-B646-094B092C6166

Serromyia mangrovi Delécole and Braverman, 1987 [Delécolle and Braverman 1987: 57]. Type locality: Egypt.

##### Distribution

PA: Egypt.

Local distribution in Egypt: Sinai: E-Shira el Gharkana, Marsa Abu Zabad, Ras Muhammad.

Dates of collection in Egypt: Unknown.

#### JOHANNSENOMYIINI

0B2515B6-69AD-515A-A103-28CA56FBB966

#### Johannsenomyia

Malloch, 1915

1788A4B1-CD34-5A1B-AC38-00F5CFB2864E

https://www.gbif.org/species/1630921

Johannsenomyia ***Johannsenomyia*** Malloch, 1915 [Malloch 1915a: 332]. Type species: *Johannsenomyia halteralis* Malloch, by subsequent designation of Wirth (1952).
Dicrohelea Kieffer, 1917 [*Kieffer 1917*: 363]. Type species: *Palpomyia filicornis* Kieffer, by subsequent designation of Macfie (1940).

#### Johannsenomyia imparunguis

(Becker, 1903)

E9F3A6F5-B7C6-5E48-81AC-36BEA4317F88

Ceratopogon imparunguis Becker, 1903 [Becker 1903: 72]. Type locality: Egypt.

##### Distribution

PA: Egypt.

Local distribution in Egypt: Lower Nile Valley & Delta: Cairo. Upper Nile Valley: Luxor.

Dates of collection in Egypt: November and December.

#### Macropeza

Meigen, 1818

7A0EBF7C-B72A-550A-9E01-813CF5246740

Macropeza ***Macropeza*** Meigen, 1818 [Meigen 1818: 87]. Type species: *Macropeza albitarsis* Meigen, by monotypy.
Macroptilum Becker, 1903 [Becker 1903: 76]. Type species: *Macroptilum nudum* Becker, by monotypy.
Haasiella Kieffer, 1913 [*Kieffer 1913c*: 190]. Type species: *Haasiella semiflava* Kieffer, by original designation.

#### Macropeza nuda

(Becker, 1903)

4959AA15-4451-5A02-95F5-10B7EDDCF67C

Macroptilum nudum Becker, 1903 [Becker 1903: 77]. Type locality: Egypt.

##### Distribution

PA: Egypt.

Local distribution in Egypt: Lower Nile Valley & Delta: Cairo. Upper Nile Valley: Sohag (EI-Shewash).

Dates of collection in Egypt: September to November.

#### Nilobezzia

Kieffer, 1921

B1B25C1F-2555-5DF1-BA31-43BB95980DCD

https://www.gbif.org/species/1630740

Nilobezzia ***Nilobezzia*** Kieffer, 1921 [Kieffer 1921a: 24]. Type species: *Nilobezzia armata* Kieffer, by monotypy.
Crespinia Kieffer, 1923 [*Kieffer 1923b*: 141]. Type species: *Crespinia brevipalpis* Kieffer, by monotypy.
Parrotia Kieffer, 1923 [*Kieffer 1923b*: 140]. Type species: *Parrotia flaviventris* Kieffer (= *Nilobezzia kiefferi* Wirth), by original designation.
Sphaerobezzia Zilahi-Sebess, 1940 [*Zilahi-Sebess 1940*: 108]. Type species: *Bezzia paradoxa* Zilahi-Sebess (= *Ceratopogon formosa* Loew), by monotypy.

#### Nilobezzia nilotica

(Kieffer, 1925)

AD2582EC-92C6-593D-A702-98FC52B01052

Parrotia nilotica Kieffer, 1925 [*Kieffer 1925c*: 263]. Type locality: Egypt (Maadi, on border of the Nile).

##### Distribution

PA: Egypt.

Local distribution in Egypt: Lower Nile Valley & Delta: Maadi.

Dates of collection in Egypt: October.

#### PALPOMYIINI

3A55870C-BC3A-5CC9-BA65-36B9D4C332DE

#### Bezzia

Kieffer, 1899

382ED726-68D2-5909-91C4-A9AF7B702954

https://www.gbif.org/species/1637599

Bezzia ***Bezzia*** Kieffer, 1899 [Kieffer 1899: 69]. Type species: *Ceratopogon ornatus* Meigen, by original designation.
Pseudobezzia Malloch, 1915 [*Malloch 1915a*: 351]. Type species: *Ceratopogon expolitus* Coquillett, by original designation.
Allobezzia Kieffer, 1917 [*Kieffer 1917*: 296]. Type species: *Ceratopogon expolitus* Coquillett, by original designation.
Lasiobezzia Kieffer, 1925 [Kieffer 1925b: 54]. Type species: *Bezzia pilipennis* Lundström, by original designation.
Homobezzia Macfie, 1932 [Macfie 1932: 496]. Type species: *Homobezzia nyasae* Macfie, by monotypy.
Sivabezzia Remm, 1974 [Remm 1974: 440]. Type species: *Bezzia campanai* Clastrier, by original designation.
Pygobezzia Remm, 1974 [*Remm 1974*: 441]. Type species: *Bezzia strobli* Kieffer (= *Ceratopogon albicornis* Meigen), by original designation.
Aspinabezzia Dow and Turner, 1976 [*Dow and Turner 1976*: 122]. Type species: *Ceratopogon glaber* Coquillett, by original designation.

#### Bezzia aegyptia

Kieffer, 1925

09C6BC41-4DD1-5AF6-8C4E-85E9411C0DFA

Bezzia aegyptia Kieffer, 1925 [*Kieffer 1925c*: 264]. Type locality: Egypt (Suez).

##### Distribution

PA: Egypt.

Local distribution in Egypt: Eastern Desert: Suez.

Dates of collection in Egypt: September.

#### Bezzia albicornis

(Meigen, 1818)

35BB09E0-4769-5ADB-B5F5-4AB51D6CAC07

Ceratopogon albicornis Meigen, 1818 [Meigen 1818: 74]. Type locality: Germany.
Ceratopogon pallidetarsata Strobl, 1900 [*Strobl 1900*Strobl, 1900: 171]. Type locality: Spain.
Bezzia strobli Kieffer, 1919 [Kieffer 1919: 122]. Type locality: Montenegro.
Bezzia brevinervis Kieffer, 1919 [Kieffer 1919: 122]. Type locality: Hungary.
Homobezzia atrata Macfie, 1944 [*Macfie 1944*: 126]. Type locality: Egypt.

##### Distribution

AF: Gambia, Nigeria, South Africa, United Arab Emirates, Yemen. PA: Afghanistan, Algeria, Egypt, Europe (widespread), Israel, Lebanon.

Local distribution in Egypt: Fayoum: Etsa, Fayoum City. Lower Nile Valley & Delta: Banha, Cairo, EI-Mansoura, El-Qanatir, Maadi, Marsafa. Western Desert: EI-Bustan (Beheira).

Dates of collection in Egypt: March to November.

## Supplementary Material

XML Treatment for LEPTOCONOPINAE

XML Treatment for Leptoconops

XML Treatment for Holoconops

XML Treatment for Leptoconops (Holoconops) kerteszi

XML Treatment for Leptoconops (Holoconops) macfiei

XML Treatment for Leptoconops (Holoconops) transversalis

XML Treatment for FORCIPOMYIINAE

XML Treatment for Atrichopogon

XML Treatment for Atrichopogon callipotami

XML Treatment for Atrichopogon flavitarsatus

XML Treatment for Atrichopogon homoius

XML Treatment for Atrichopogon isis

XML Treatment for Atrichopogon luteicollis

XML Treatment for Atrichopogon osiris

XML Treatment for Forcipomyia

XML Treatment for Euprojoannisia

XML Treatment for Forcipomyia (Euprojoannisia) psilonota

XML Treatment for Forcipomyia

XML Treatment for Forcipomyia (Forcipomyia) biannulata

XML Treatment for Forcipomyia (Forcipomyia) nilicola

XML Treatment for Forcipomyia (Forcipomyia) urnigera

XML Treatment for Lepidohelea

XML Treatment for Forcipomyia (Lepidohelea) pulcherrima

XML Treatment for Microhelea

XML Treatment for Forcipomyia (Microhelea) fuliginosa

XML Treatment for Synthyridomyia

XML Treatment for Forcipomyia (Synthyridomyia) murina

XML Treatment for DASYHELEINAE

XML Treatment for Dasyhelea

XML Treatment for Dasyhelea arenivaga

XML Treatment for Dasyhelea arenosa

XML Treatment for Dasyhelea begueti

XML Treatment for Dasyhelea egypti

XML Treatment for Dasyhelea fusca

XML Treatment for Dasyhelea heliophila

XML Treatment for Dasyhelea ismailiae

XML Treatment for Dasyhelea luteocincta

XML Treatment for Dasyhelea modesta

XML Treatment for Dasyhelea nyasae

XML Treatment for CERATOPOGONINAE

XML Treatment for CULICOIDINI

XML Treatment for Culicoides

XML Treatment for Culicoides azerbajdzhanicus

XML Treatment for Culicoides bedfordi

XML Treatment for Culicoides calloti

XML Treatment for Culicoides cataneii

XML Treatment for Culicoides catharinae

XML Treatment for Culicoides circumscriptus

XML Treatment for Culicoides distinctipennis

XML Treatment for Culicoides firuzae

XML Treatment for Culicoides hanae

XML Treatment for Culicoides imicola

XML Treatment for Culicoides jumineri

XML Treatment for Culicoides kingi

XML Treatment for Culicoides langeroni

XML Treatment for Culicoides leucostictus

XML Treatment for Culicoides marcleti

XML Treatment for Culicoides maritimus

XML Treatment for Culicoides mesghalii

XML Treatment for Culicoides navaiae

XML Treatment for Culicoides neavei

XML Treatment for Culicoides newsteadi

XML Treatment for Culicoides pilosipennis

XML Treatment for Culicoides pulicaris

XML Treatment for Culicoides puncticollis

XML Treatment for Culicoides riethi

XML Treatment for Culicoides sahariensis

XML Treatment for Culicoides schultzei

XML Treatment for Culicoides sejfadinei

XML Treatment for Culicoides sergenti

XML Treatment for Culicoides similis

XML Treatment for Culicoides stigma

XML Treatment for Culicoides vitreipennis

XML Treatment for CERATOPOGONINI

XML Treatment for Alluaudomyia

XML Treatment for Alluaudomyia melanosticta

XML Treatment for Serromyia

XML Treatment for Serromyia mangrovi

XML Treatment for JOHANNSENOMYIINI

XML Treatment for Johannsenomyia

XML Treatment for Johannsenomyia imparunguis

XML Treatment for Macropeza

XML Treatment for Macropeza nuda

XML Treatment for Nilobezzia

XML Treatment for Nilobezzia nilotica

XML Treatment for PALPOMYIINI

XML Treatment for Bezzia

XML Treatment for Bezzia aegyptia

XML Treatment for Bezzia albicornis

## Figures and Tables

**Table 1.** Taxa treated in the catalogue.

**Subfamily**	**Tribes**	**Genera**	**Species**
Leptoconopinae	—	1	**3**
Forcipomyiinae	—	2	**13**
Dasyheleinae	—	1	**10**
Ceratopogoninae	4	7	**38**
**Total**	**4**	**11**	**64**

## References

[B5576498] Abdel-Samei M. (1974). Ecological studies on *Culicoides* in A. R. Egypt, M.D. Thesis.

[B5576507] Ahmed S. (1991). A computerized approach towards the taxonomy of blood sucking flies except mosquitoes in Egypt. Ph.D. Thesis.

[B5576536] Alwin-Kownacka Alicja, Szadziewski Ryszard, Szwedo Jacek (2016). Biting midges of the subfamily Forcipomyiinae (Diptera: Ceratopogonidae) from the Middle East, with keys and descriptions of new species. Zootaxa.

[B5576546] Alwin-Kownacka Alicja, Szadziewski Ryszard, Szwedo Jacek (2017). Predatory midges of the tribes Palpmyiini and Sphaeromiini (Diptera: Ceratopogonidae) from the Middle East, with keys and descriptions of new species. European Journal of Taxonomy.

[B5576566] Austen Ernest E. (1912). Notes on African blood-sucking midges (Family Chironomidae, Sub-family Ceratopogoninae), with descriptions of new species. Bulletin of Entomological Research.

[B5576576] Austen E. (1921). A Contribution to Knowledge of the Blood-sucking Diptera of Palestine, other than Tabanidae. Bulletin of Entomological Research.

[B5576596] Becker T. (1903). Ägyptische Dipteren. Mitteilungen aus dem Zoologischen Museum.

[B5576606] Becker T. (1908). Dipteren der Kanarischen Inseln. Mitteilungen aus dem Zoologischen Museum.

[B5576626] Bellis G., Dyce A. (2011). Marksomyia, a new subgenus of *Culicoides* Latreille (Diptera: Ceratopogonidae) from the Australasian biogeographic region with descriptions of two new species. Zootaxa.

[B5576636] Bigot J. (1857). Essai d’une classification générale et synoptique de l’ordre des Insectes Diptères. 5e Mémoire. Annales de la Société Entomologique de France.

[B5576759] Boorman J., Lane R. P. (1979). A new genus and species of Ceratopogonidae (Diptera) closely related to *Culicoides* from West Africa. Journal of Natural History.

[B5576679] Boorman John (1993). Biting midges (Ceratopogonidae). Medical Insects and Arachnids.

[B5576733] Boorman J., van Harten A. (2002). Some Ceratopogonidae (Insecta: Diptera) from the Arabian Peninsula, with particular reference to the Republic of Yemen. Fauna of Arabia.

[B5725803] Borkent A., Wirth WW (1997). World species of biting midges (Diptera: Ceratopogonidae). Bulletin of the AmericanMuseum of Natural History.

[B5576779] Borkent A. World species of biting midges (Diptera: Ceratopogonidae). http://www.inhs.illinois.edu/research/FLYTREE/CeratopogonidaeCatalog.pdf.

[B5576815] Braverman Y., Delécolle J. C., Frish K., Rubina M., Kremer M. (1981). New records of *Culicoides* species (Diptera: Ceratopogonidae) from Golan Heights, Israel and Sinai Peninsula. Israel Journal of Entomology.

[B5576895] Braverman Y., Delecolle J. C., Kremer M. (1983). Culicoides (Oecacta) hanae (Diptera: Ceratopogonidae), a new species from Israel and Sinai. Journal of Medical Entomology.

[B5576905] Brèthes J. (1912). Descripcion de un nuevo género y especie nueva de Chironomidae (Dipt). Anales del Museo Nacional de Buenos Aires.

[B5576915] Brèthes J. (1914). Descripcion de six Cécidomyidae (Dipt.) de Buenos Aires. Anales del Museo Nacional de Buenos Aires.

[B5577003] Brunetti Enrico. (1920). Catalogue of Oriental and south Asiatic Nemocera / by E. Brunetti.. Records of the Indian Museum.

[B5577045] Callot J., Kremer M., Deduit Y. (1962). Nouvelles espèces et nouvelles localisations de *Culicoides* (Diptera: Ceratopogonidae) des Ardennes, du Centre de la France, du Jura français et du Jura suisse. Annales de Parasitologie Humaine et Comparée.

[B5577035] Callot J., Kremer M., Basset M. (1968). *Culicoides
marcleti* n. sp. et nouvelles localisations de *Culicoides* (Diptères, Cératopogonidés) de la région méditerranéenne et particulièrement d'Algerie. Bulletin de la Société de Pathologie Exotique.

[B5577013] Callot J., Kremer M. (1969). Description d'un Culicoide nouveau *C.
jumineri* (Dipt. Cératopogonidé) trouvé en Tunisie. Bulletin de la Société de Pathologie Exotique.

[B5577025] Callot J., Kremer M., Bailly-Choumara H. (1970). Description de *Culicoides
coluzzii* n. sp. (Dipt., Ceratopogonidae). Bulletin de la Société Zoologique de France.

[B5577055] Carter Henry F. (1916). On three new African midges. Annals of Tropical Medicine & Parasitology.

[B5577146] Carter Henry F. (1919). New West African Ceratopogoninae. Annals of Tropical Medicine & Parasitology.

[B5577166] Carter Henry F., Ingram A., Macfie J. W. S. (1920). Observations on the ceratopogonine midges of the Gold Coast with descriptions of new species. Annals of Tropical Medicine & Parasitology.

[B5577156] Carter Henry F. (1921). A revision of the genus *Leptoconops* Skuse. Bulletin of Entomological Research.

[B5577207] Carter Henry F., Ingram A., Macfie J. W. S. (1921). Observations on the ceratopogonine midges of the Gold Coast with descriptions of new species. Part III. Annals of Tropical Medicine & Parasitology.

[B5577177] Carter Henry F., Ingram A., Macfie J. W. S. (1921). Observations on the ceratopogonine midges of the Gold Coast with descriptions of new species. Part IV. Annals of Tropical Medicine & Parasitology.

[B5577217] Choufani J., Perrichot V., Girard V., Garrouste R., Azar D., Nel A. (2013). Two new biting midges of the modern type from Santonian amber of France (Diptera: Ceratopogonidae). Insect Evolution in an Amberiferous and Stone Alphabet. Arthropods and Amber. E.J. Brill, Leiden,.

[B5577253] Chu F. I. (1983). Two new subgenera and two new species of *Culicoides* from China (Diptera: Ceratopogonidae). Entomotaxonomia.

[B5577286] Clastrier J. (1957). Notes sur les Cératopogonidés. II. Quelques *Culicoides* à ailes tachetées. Archives de l'Institut Pasteur Algérie.

[B5577296] Clastrier J. (1959). Notes sur les Cératopogonidés. VII. Cératopogonidés d'Afrique Occidentale Francaise (4). Archives de l'Institut Pasteur Algérie.

[B5577328] Clastrier J. (1975). Le genre Leptoconops, sous-genre Holoconops en Afrique du Nord (Diptera, Ceratopogonidae). Archives de l'Institut Pasteur Algérie.

[B5577342] Coquillett D. W. (1910). The type-species of the North American genera of Diptera. Proceedings of the United States National Museum.

[B5577353] Cordero E. H. (1929). Contribución al estudio de los Dipteros del Uruguay, I. *Lophomyidium
uruguayense* n. gen., n. sp. Nueva Ceratopogonina hematófaga. Anales del Museo de Historia Natural de Montevideo.

[B5651736] Das Gupta S. K. (1962). Some *Culicoides* of Calcutta and the neighbouring areas. Science and Culture. Science and Culture.

[B5651855] Delécolle J. C., Braverman Y. (1987). Description de *Serromyia
mangrovi* n. sp. du Sinai (Dipt. Ceratopogonidae). Bulletin de la Société Entomologique de France.

[B5577363] De Meijere Johannes Cornelis Hendrik de. (1907). Studien über südostasiatische Dipteren. I.. Tijdschrift voor Entomologie.

[B5577385] De Meillon Botha (1939). Notes on Ceratopogonidae (Dipt. Nematocera) from southern Africa. Journal of the Entomological Society of Southern Africa.

[B5577396] De Meillon B. (1943). New records and new species of Nematocera (Diptera) from the Ethiopian Region. Journal of the Entomological Society of Southern Africa.

[B5577416] Dominiak PATRYCJA, Szadziewski RYSZARD (2010). Distribution and new synonymy in European biting midges of the genus *Dasyhelea* Kieffer (Diptera: Ceratopogonidae). Zootaxa.

[B5577438] Dow M. I., Turner E. C. (1976). A revision of the Nearctic species of the genus *Bezzia* (Diptera: Ceratopogonidae). Research Division Bulletin, Virginia Polytechnic Institute and State University.

[B5577448] Downes J. A. (1978). Feeding and mating in the insectivorous Ceratopogoninae (Diptera). Memoirs of the Entomological Society of Canada.

[B5577458] Dyce AL, Meiswinkel R (1995). Tokunagahelea, a new subgenus of *Culicoides* Latreille (Diptera: Ceratopogonidae) from the Australasian region with descriptions of two new species. Invertebrate Systematics.

[B5577468] Dzhafarov S. M. (1958). New species of biting flies (Diptera, Heleidae) from Nakhichevanskoi ASSR. Doklady Akademii Nauk Azerbaidzhanskoi SSR.

[B5577478] Dzhafarov S. M. (1959). *Culicoides
flavidus*, a new species of the genus *Culicoides* Kieff. (Diptera, Heleidae) from Transcaucasia. Entomologicheskoe Obozrenie.

[B5577488] Dzhafarov S. M. (1962). New species of bloodsucking midges (Diptera, Heleidae) from the valley of the Kura River, Transcaucasus. Entomologicheskoe Obozrenie.

[B5577498] Dzhafarov S. M. (1964). Blood-sucking midges (Diptera, Heleidae) of the Transcaucasus. Akademija Nauk Azerbaidzanskoi SSR, Instituta Zoologicheskiy, Russia,.

[B5577518] Edwards F. W. (1929). Philippine nematocerous Diptera II.. Notulae Entomologicae.

[B5577528] Eesa N. M. (1975). The systematics and bionomics of *Culicoides* in Egypt. M.Sc. Thesis.

[B5577606] El-Hawagry Magdi, Gilbert Francis (2014). Zoogeographical affinities and faunal relationships of bee flies (Diptera: Bombyliidae) in Egypt. Zoology in the Middle East.

[B5577549] El-Hawagry MAGDI S. (2017). Catalogue of Egyptian Tephritoidea (Diptera: Schizophora: Acalyptratae). Zootaxa.

[B5577560] El-Hawagry Magdi S. (2018). Catalogue of the Tachinidae of Egypt (Diptera: Oestroidea). Egyptian Journal of Biological Pest Control.

[B5577925] El-Hawagry MAGDI S., Zatwarnicki TADEUSZ, Ebrahim AYMAN M. (2018). Catalogue of the Egyptian Ephydroidea (Diptera: Schizophora: Acalyptratae). Zootaxa.

[B5577574] El-Hawagry M., El-Azab S. A. (2019). Catalog of the Calliphoridae, Rhiniidae, and Sarcophagidae of Egypt (Diptera: Oestroidea). Egyptian Journal of Biological Pest Control.

[B5577915] El-Hawagry MAGDI S., Gilbert FRANCIS (2019). Catalogue of the Syrphidae of Egypt (Diptera). Zootaxa.

[B5577594] El-Hawagry M., El-Azab S., Gilbert F. (2019). Catalogue of the family Pipunculidae in Egypt (Diptera: Cyclorrhapha). African Entomology.

[B5577584] El-Hawagry Magdi, Ebrahim Ayman Mohey Eldin, Nada Maha Salah Eldin (2020). First detection of the Nearctic parasitoid species *Trichopoda
pennipes* (Fabricius) (Diptera: Tachinidae) in Egypt. Egyptian Journal of Biological Pest Control.

[B5576479] EL-Hawagry M. (2015). Catalogue of superfamily Asiloidea.

[B5577935] Enderlein G. (1908). Neue Ceratopogoninen aus Südafrika. Denkschriften der medizinisch-naturwissenschaftlichen Gesellschaft zu Jena.

[B5577945] Enderlein G., Brohmer P., Ehrmann P., Ulmer G. (1936). Ordnung Zweiflügler, Diptera. Die Tierwelt Mitteleuropas 6: Insekten.

[B5577959] Evenhuis NEAL L., Pont ADRIAN C. (2004). The Diptera Genera of Jacques-Marie-Frangile Bigot. Zootaxa.

[B5733925] FAO World Development Indicators: Average Precipitation in Depth (mm per year). http://data.worldbank.org/indicator/AG.LND.PRCP.MM.

[B5585605] Filatov Serhii, Szadziewski Ryszard (2017). Annotated checklist and distribution of *Culicoides* biting midges of Ukraine (Diptera: Ceratopogonidae). Journal of Natural History.

[B5577969] Fox I. (1948). *Hoffmania*, a new subgenus in *Culicoides* (Diptera: Ceratopogonidae). Proceedings of the Biological Society of Washington.

[B5577979] Fox I. (1955). A catalogue of the bloodsucking midges of the Americas (Culicoides, Leptoconops and Lasiohelea) with keys to the subgenera and Nearctic species, a geographic index and bibliography. Journal of Agriculture of the University of Puerto Rico.

[B5577989] Garrett C. B.D. (1925). Seventy new Diptera.

[B5578017] Ghonaim Mohamed F., Ibrahim Abdelwahab A., Ali Arshad (2001). A review of the genus *Forcipomyia* (Diptera: Ceratopogonidae) from Egypt with description of a new species. Oriental Insects.

[B5578007] Ghonaim Mohamed F., Fadl Hassan H., Ibrahim Abdel Wahab A., Ali Arshad (2001). An annotated checklist of the Ceratopogonidae (Diptera) of Egypt. Oriental Insects.

[B5577998] Ghonaim M. F. (1997). Ecological and taxonomic studies on the biting midges (Diptera: Ceratopogonidae) in Egypt. M.Sc. Thesis.

[B5578027] Glukhova V. M. (1977). The subgeneric classification of the genus *Culicoides* Latreille, 1809 (Diptera, Ceratopogonidae), with a consideration of the structure of the larval phase. Parazitologicheskii Sbornik.

[B5578037] Glukhova V. M. (1989). Blood-sucking midges of the genera *Culicoides* and *Forcipomyia* (Ceratopogonidae). Fauna of the USSR 139.

[B5578047] Goeldi E. (1905). Os mosquitos no Pará. Reunião de quatro trabalhos sobre os mosquitos indigenas, principalmente as especies que molestam o homem. Memorias do Museu Goeldi (Museu Paraense) de Historia Natural e Ethnographie.

[B5578057] Goetghebuer M. (1910). Description de diptères chironomides nouveaux. Revue Mensuelle de la Société Entomologique Namuroise.

[B5578067] Goetghebuer Maurice. (1920). Ceratopogoninae de Belgique, par le dr M. Goetghebuer.. Mémoires du Musée Royal d'Histoire Naturelle de Belgique.

[B5578077] Goetghebuer Maurice. (1921). Chironomides de Belgique et spécialement de la zone des Flandres. Mémoires du Musée Royal d'Histoire Naturelle de Belgique.

[B5578087] Goetghebuer M. (1933). Catalogue des Cératopogonides de Belgique. Bulletin & Annales de la Société Entomologique de Belgique.

[B5578098] Goetghebuer M. (1933). Ceratopogonidae et Chironomidae du Congo Belge. Revue de Zoologie et de Botanique Africaines.

[B5578109] Goetghebuer M., Lindner E. (1933). 13a. Heleidae (Ceratopogonidae). Die Fliegen der palaearktischen Region.

[B5578123] Goetghebuer M. (1935). Cératopogonides récoltés par le Dr. De Wulf au Congo Belge. Revue de Zoologie et de Botanique Africaines.

[B5578133] Goetghebuer M. (1948). Ceratopogonidae (Diptera
Nematocera). Exploration du Parc National Albert I. Mission G.F. De Witte 1933-1935.

[B5578143] Goetghebuer M. (1950). Ceratopogonidae et Chironomidae nouveaux ou peu connus d'Europe (Quatorzieme note). Bulletin Institut Royal des Sciences Naturelles de Belgique.

[B5578153] Gutsevich A. V. (1959). New species of the genus *Culicoides* (Diptera, Heleidae) from the southern regions of the USSR. Entomologicheskoe Obozrenie.

[B5578163] Harant H., Baur O. (1946). *Lasiohelea
wansoni* n. sp. Cératopogonide du Congo belge. Archives de l'Institut Pasteur Algérie.

[B5578173] Ingram A., Macfie J. W. S. (1921). West African Ceratopogoninae. Annals of Tropical Medicine & Parasitology.

[B5578183] Ingram A., Macfie J. W. S. (1922). West African Ceratopogoninae Part II. Annals of Tropical Medicine & Parasitology.

[B5578193] Ingram A., Macfie J. W.S. (1923). Notes on some African Ceratopogoninae. Bulletin of Entomological Research.

[B5578203] Ingram A., Macfie J. W. S. (1924). Notes on some African Ceratopogoninae—species of the genus *Forcipomyia*. Annals of Tropical Medicine & Parasitology.

[B5578213] Ingram A., Macfie J. W. S. (1925). New Ceratopogoninae from Nyasaland (Dipt.). Bulletin of Entomological Research.

[B5578223] Karsch F. (1886). Ein neues märkisches Dipteron (*Ceratopogon
crudelis* n. sp.). Berliner Entomologische Zeitschrift.

[B5578233] Khalaf Kamel (1954). The speciation of the genus *Culicoides* (Diptera, Heleidae). Annals of the Entomological Society of America.

[B5578243] Khalaf K. T. (1957). Heleids from Iraq, with description of new species (Diptera: Heleidae (Ceratopogonidae)). Bulletin de la Société Entomologique d'Egypte.

[B5578253] Kieffer J J (1899). Description d'un nouveau genre et tableau des genres européens de la famille des chironomides (Dipt.). Bulletin de la Société entomologique de France..

[B5578263] Kieffer J. J., Wytsman P. (1906). Diptera. Fam. Chironomidae. Genera Insectorum.

[B5578277] Kieffer J. J. (1908). Description d'une espèce nouvelle de chironomides d'Égypte. Annales Historico-Naturales Musei Nationalis Hungarici.

[B5578297] Kieffer J. J. (1911). The Percy Sladen Trust Expedition to the Indian Ocean in 1905. Under the leadership of Mr. J. Stanley Gardiner. Vol. 3. No. XV. Diptera, Chironomidae der Seychellen-inseln, aus der Sammlung von Mr. H. Scott. Transactions of the Linnean Society of London (2nd Ser.).

[B5578307] Kieffer J. J. (1911). Nouvelles descriptions de chironomides obtenus d'éclosion. Bulletin de la Société d'Histoire Naturelle de Metz.

[B5578317] Kieffer J J (1912). Nouveaux chironomides (Tendipedidae) de Ceylan. Spolia zeylanica..

[B5578327] Kieffer J. J. (1913). Nouveaux chironomides (tendipédides) d'Allemagne. Bulletin de la Société d'Histoire Naturelle de Metz.

[B5578337] Kieffer J. J. (1913). Nouvelle contribution a la connaissance des tendpédides d'Allemagne. Bulletin de la Société d'Histoire Naturelle de Metz.

[B5578357] Kieffer J. J. (1913). Nouvelle etude sur les chironomides de l'Indian Museum de Calcutta. Records of the Indian Museum.

[B5578377] Kieffer J. J., Alluaud C. A., Jeannel R. (1913). Chironomidae et Cecidomyidae. Voyage de Ch. Alluaud et R. Jeannel en Afrique orientale (1911-1912). Resultats scientifiques. Diptera.

[B5578391] Kieffer J. J. (1914). Zwölf neue Culicoidinenarten. Archiv für Hydrobiologie und Planktonkunde, Supplement.

[B5578401] Kieffer J J (1914). South African Chironomidae (Diptera). Annals of the South African Museum. Annale van die Suid-Afrikaanse Museum..

[B5578411] Kieffer J. J. (1915). Neue halophile Chironomiden. Archiv für Hydrobiologie, Supplement.

[B5578421] Kieffer J. J. (1917). Chironomides d'Amérique conservés au Musée National Hongrois de Budapest. Annales Historico-Naturales Musei Nationalis Hungarici.

[B5578431] Kieffer J. J. (1918). Chironomides d'Afrique et d'Asie conservés au Museum National Hongrois de Budapest. Annales HistoricoNaturales Musei Nationalis Hungarici.

[B5578441] Kieffer J. J. (1918). Beschreibung neuer, auf Lazarettschiffen des östlichen Kriegsschauplatzes und bei Ignalino in Litauen von Dr. W. Horn gesammelter Chironomiden, mit Uebersichtstabellen einiger Gruppen von paläarktischen Arten (Dipt.). Entomologische Mitteilungen 7: 35-53, 94-110, 163-170, 177-188.

[B5578451] Kieffer J. J. (1919). Chironomides d'Europe conservés au Musée National Hongrois de Budapest. Annales Historico-Naturales Musei Nationalis Hungarici.

[B5578461] Kieffer J. J. (1921). Chironomides de l'Afrique Équatoriale. Annales de la Société Entomologique de France.

[B5578471] Kieffer J. J. (1921). Sur quelques Diptères piqueurs de la tribu des Ceratopogoninae. Archives de l'Institut Pasteur de l'Afrique du Nord.

[B5578481] Kieffer J. J. (1921). Nouvelles observations sur les Diptères piqueurs de la tribu des Ceratopogoninae. Archives de l'Institut Pasteur de l'Afrique du Nord.

[B5578491] Kieffer J. J. (1922). Étude sur les Chironomides de Formose. Annales de la Société linnéenne de Lyon.

[B5578501] Kieffer J. J. (1922). Nouveaux Chironomides piqueurs habitant l'Algérie. Archives de l'Institut Pasteur de l'Afrique du Nord.

[B5578511] Kieffer J. J. (1923). Ceratopogonines recueillis au Sahara Constantinois. Archives de l'Institut Pasteur Algérie.

[B5578521] Kieffer J. J. (1923). Chironomides piqueurs de Java. Annales de la Société Scientifique de Bruxelles.

[B5578531] Kieffer J. J. (1924). Chironomides nouveaux ou rares de l'Europe centrale. Bulletin de la Société d'Histoire Naturelle de la Moselle.

[B5578541] Kieffer J. J. (1924). Quelques nouveaux chironomides piqueurs de l'Europe centrale. Archives de l'Institut Pasteur Algérie.

[B5578551] Kieffer J. J. (1925). Nouveaux genres et nouvelles espèces de chironomides piqueurs. Archives de l'Institut Pasteur Algérie.

[B5578561] Kieffer J. J. (1925). Diptères (Nématocères piqueurs): Chironomidae
Ceratopogoninae. Faune de France.

[B5578571] Kieffer J. J. (1925). Chironomides d'Egypte (Dipt.). Bulletin de la Société Royale Entomologique d'Egypte.

[B5730939] Kirk-Spriggs AH, Sinclair BJ (2017). Manual of Afrotropical Diptera, Introductory chapters and keys to Diptera families.

[B5578581] Kono H., Takahasi H. (1940). A revision of the *Culicoides*-species of Saghalien and Hokkaido (Ceratopogonidae, Diptera). Insecta Matsumurana.

[B5578601] Kremer M., Delécolle J. C., Bailly-Choumara H., Chaker E. (1979). Cinquième contribution à l'étude faunistique des *Culicoides* (Diptera, Ceratopogonidae) du Maroc description de *C.
calloti* n. sp. Cahiers ORSTOM, Série Entomologie Médicale et Parasitologie.

[B5578611] Kremer M., Delécolle J. C., Braverman Y. (1991). A new and a redescribed species of *Culicoides* from Sinai (Diptera: Ceratopogonidae). Israel Journal of Zoology 37: 151-157..

[B5578621] Lane R. P. (1983). Insects of Saudi Arabia *Culicoides* (Diptera: Ceratopogonidae) of Saudi Arabia and their potential veterinary importance. Fauna of Saudi Arabia.

[B5578641] Latreille P. A. (1809). Genera crustaceorum et insectorum secundum ordinem naturalem in familias disposita, iconibus exemplisque plurimis explicata.

[B5578650] Lee T. S. (1982). Diptera: Ceratopogonidae. The Series of the Comprehensive Scientific Expedition to the Qinghai-Xizang Plateau. Insects of Xizang.

[B5578674] Lien J. C., Weng M. H., Lin C. C. (1996). Biting midges of the genus *Culicoides* (Diptera, Ceratopogonidae) from Nankan Is. of the Matsu area, with description of two new species. Chinese Journal of Parasitology.

[B5578664] Linley John R., Davies John B. (1971). Sandflies and tourism in Florida and the Bahamas and Caribbean Area 1. Journal of Economic Entomology.

[B5578995] Linnaeus C. (1758). Systema naturae per regna tria naturae, secundum classes, ordines, genera, species, cum characteribus, differentiis, synonymis, locis.

[B5579004] Liu J. H., Yan G., Liu G. P., Hao B. S., Liu Z. J., Yu Y. X. (2001). Forcipomyiinae of China (Diptera: Ceratopogonidae) I. General introduction and the genus *Atrichopogon* Kieffer. Fauna of China.

[B5579016] Lundström C. (1910). Beiträge zur Kenntnis der Dipteren Finlands VI. Chironomidae. Acta Societatis pro Fauna et Flora Fennica.

[B5579046] Macfie J. W.S. (1932). LII.—Some new or little-known Ceratopogonidæ. Annals and Magazine of Natural History.

[B5579026] Macfie J. W. S. (1924). On some Egyptian Certopogoninae. Bulletin of Entomological Research.

[B5579036] Macfie J. W. S. (1925). A new blood-sucking midge from Singapore. Bulletin of Entomological Research.

[B5579066] Macfie J. W. S. (1940). The genera of Ceratopogonidae. Annals of Tropical Medicine & Parasitology.

[B5579076] Macfie J. W. S. (1943). Ceratopogonidae (Diptera) from Egypt. Proceedings of the Royal Entomological Society of London. Series B, Taxonomy.

[B5579086] Macfie J. W. S. (1944). A new species of *Homobezzia* (Diptera, Ceratopogonidae) from Egypt. Proceedings of the Royal Entomological Society of London. Series B, Taxonomy.

[B5579096] Malloch John Russell (1915). The Chironomidae, or midges, of Illinois, with particular reference to the species occurring in the Illinois River. Bulletin of the Illinois State Laboratory of Natural History.

[B5579106] Malloch J. R. (1915). Some additional records of Chironomidae for Illinois and notes on other Illinois Diptera. Bulletin of the Illinois State Laboratory of Natural History.

[B5579116] Mayer K. (1937). Beobachtunen über blutsaugende Ceratopogoniden. Arbeiten über morphologische und taxonomische Entomologie aus Berlin-Dahlem.

[B5579136] Meigen J. W. (1818). Systematische Beschreibung der bekannten europaischen zweiflugeligen Insekten.

[B5579145] Mirzaeva A. G., Isaev V. A. (1990). Revision of the subgenus
Culicoides s. str. (Diptera, Ceratopogonidae). Novye I Maloizvetnye Vidy Fauny Sibiri.

[B5579155] Morsy T. A. (1961). Taxonomic and biological studies on *Culicoides*. M.Sc. Thesis, Faculty of Medicine, Ain Shams University, Egypt, 242 pp..

[B5579164] Morsy T. A., Bebars M. A., Sabry A. A., Ahmed M. M., Abdel Fattah S. A. (1989). Studies on biting midges of the genus *Culicoides* in the Suez Canal Zone. Journal of the Egyptian Society of Parasitology.

[B5579175] Nagaty H. F., Morsy T. A. (1959). *Culicoides
pallidipennis*, C., L. & M. (Diptera: Ceratopogonidae), an Egyptian biting midge. Journal of the Egyptian Public Health Association.

[B5579185] Nagaty H. F., Morsy T. A. (1959). *Culicoides
puncticollis* (Becker) (Diptera: Ceratopogonidae) from Egypt. Journal of the Egyptian Public Health Association.

[B5579195] Nagaty H. F., Morsy T. A. (1960). Egyptian biting midges of the genus *Culicoides* Latreille (Diptera: Chironomidae - Ceratopogonidae) with 3 text figs. Bulletin of the Entomological Society of Egypt.

[B5579206] Nagaty H. F., Morsy T. A. (1960). Report on a collection of biting midges of the genus *Culicoides* (Diptera: Ceratopogonidae). Journal of the Egyptian Public Health Association.

[B5731453] Nasser Moahmed, El-Hawagry Magdi, Okely Mohamed (2019). Environmental niche modeling for some species of the genus Anthrax Scopoli (Diptera: Bombyliidae) in Egypt, with special notes on St. Catherine protected area as a suitable habitat. Journal of Insect Conservation.

[B5579238] Navai S. (1973). *Culicoides* (Diptera: Ceratopogonidae) from the Persian Gulf area of Iran. Part I: Two new species *C.
mesghalii* and *C.
shahgudiani*. Bulletin de la Société de Pathologie Exotique.

[B5579248] Noè G. (1905). Un nuovo genere appartenente alla famiglia Chironomidae. Rendiconti, Accademia Nazionale dei Lincei.

[B5731568] Oka H, Asahina S (1948). *Pterobosca* from Japan and her adjacent territories (Diptera, Ceratopogonidae). Mushi.

[B5579258] Pape THOMAS, Blagoderov VLADIMIR, Mostovski MIKHAIL (2011). Order Diptera Linnaeus, 1758. In: Zhang ZQ (Ed.) Animal biodiversity: An outline of higher-level classification and survey of taxonomic richness. Zootaxa.

[B5579268] Philippi R. A. (1865). Aufzahlung der chilenischen Dipteren. Verhandlungen der Kaiserlich-Königlichen Zoologisch-Botanischen Gesellschaft in Wien.

[B5579278] Poey Felipe (1853). Memórias sobre la historia natural de la Isla de Cuba, acompañadas de sumarios latinos y extractos en francés. Vol. 1. pt. 4. Havanna,.

[B5579306] Rafinesque C. S. (1815). Analyse de la nature ou tableau de l'univers et des corps organisés. Le nature est mon guide, et Linnéus mon maitre.

[B5582963] Rawlings Peter (1996). A key, based on wing patterns of biting midges (genus *Culicoides* Latreille - Diptera: Ceratopogonidae) in the Iberian Peninsula, for use in epidemiological studies. Graellsia.

[B5585555] Remm H., Zhogolev D. T. (1968). Contributions to the fauna of biting midges (Diptera, Ceratopogonidae) of the Crimea. Entomologicheskoe Obozrenie.

[B5585490] Remm H. (1974). A review of species of the genus *Bezzia* Kieffer (Diptera, Ceratopogonidae) from the fauna of the USSR. I. Entomologicheskoe Obozrenie.

[B5585520] Remm H. (1979). Eesti NSV habesääsklaste (Diptera, Ceratopogonidae) fauna kataloog. In Dipteroloogilisi Uurimusi (Tartu). Eesti NSV Teaduste Akadeemia Eesti Looduseuurijate selts,.

[B5585530] Remm H. (1980). New species of the family Ceratopogonidae (Diptera) from the Middle Asia Tartu Riikliku Ulikooli Toimetised 516: 85-128.. Tartu Riikliku Ulikooli Toimetised.

[B5585540] Remm H., Soos Á., Papp (1988). Family Ceratopogonidae. Catalogue of Palaearctic Diptera.

[B5585565] Root FRANCIS METCALF, Hoffman W. A. (1937). The North American species of *Culicoides*. American Journal of Epidemiology.

[B5585575] Santos Abreu Elias. (1918). Ensayo de una monografia de los tendipedidos de las islas canarias. Memorias de la Real Academia de Ciencias y Artes de Barcelona.

[B5585585] Saunders L. G. (1957). Revision of the genus *Forcipomyia* based on characters of all stages (Diptera, Ceratopogonidae). Canadian Journal of Zoology.

[B5585595] Sen Purnendu, Das Gupta S. K. (1959). Studies on Indian *Culicoides* (Ceratopogonidae: Diptera). Annals of the Entomological Society of America.

[B5585615] Shevchenko A. K. (1977). Bloodsucking midges. Fauna Ukraini.

[B5585625] Skuse F A A (1889). Diptera of Australia. Part VI. The Chironomidae. Proceedings of the Linnean Society of New South Wales..

[B5585636] Stephens J. F. (1829). The nomenclature of British insects; being a compendious list of such species as are contained in the Systematic Catalogue of British Insects, and forming a guide to their classification.

[B5585654] Stephens J. F. (1829). A systematic catalogue of British insects: being an attempt to arrange all the hitherto discovered indigenous insects in accordance with their natural affinities.

[B5591838] Steyskal G. C., El-Bialy S. (1967). A list of Egyptian Diptera with a bibliography and key to families. Ministry of Agriculture Technical Bulletin.

[B5591848] Strand E. (1928). Miscellanea nomenclatorica zoologica et paleontologica I-II. Archiv fur Naturgeschichte.

[B5591858] Strobl B. G. (1900). Spanische Dipteren. Wiener Entomologische Zeitung.

[B5591868] Szadziewski R. (1984). Redescriptions of three species of the biting midges Ceratopogonidae (Diptera) described by Becker from Egypt. Polish Journal of Entomology.

[B5730500] Szadziewski Ryszard (2018). Biting midges (Diptera: Ceratopogonidae) as indicators of biostratigraphy, ecological reconstructions and identification of amber deposits. Earth and Environmental Science Transactions of the Royal Society of Edinburgh.

[B5591878] Tokunaga M. (1940). Biting midges from Japan and neighbouring countries, including Micronesian Islands, Manchuria, North China and Mongolia (Diptera, Ceratopogonidae). 3: 58-100.. Tenthredo.

[B5591888] Tokunaga M. (1941). Biting midges from Manchuria (Ceratopogonidae, Diptera). Insecta Matsumurana.

[B5591898] Tokunaga M. (1963). Some Japanese biting midges breeding in paddy-field water (Diptera, Ceratopogonidae). 15: 37-49.. Scientific Reports of the Kyoto Prefectural University, Agriculture.

[B5591908] Townsend C. H. Tyler (1893). An interesting blood-sucking gnat of the family Chironomidae. Psyche: A Journal of Entomology.

[B5591918] Vargas L. (1953). Beltranmyia
n.
subgen.
de
Culicoides (Insecta: Heleidae).. Revista del Instituto de Salubridad y Enfermedades, Tropicales.

[B5591928] Vargas L. (1960). The subgenera of *Culicoides* of the Americas (Diptera, Ceratopogonidae). Revista de Biologia Tropical.

[B5591948] Vargas L., Kremer M. (1972). Callotia
n.
subg.
of
Culicoides (Diptera, Ceratopogonidae).. Mosquito News.

[B5591938] Vargas L. (1973). *Wirthomyia*, a new subgenus of *Culicoides* (Diptera: Ceratopogonidae).. Mosquito News.

[B5591958] Vimmer A. (1932). Nové druhy podceledi Ceratopogoninae (Tendipedidae—Dipt.) ze sberu Bodenheimerova. Sbornik Entomologickeho Oddeleni pri Zoologickych Sbirkach Narodniho Musea v Praze.

[B5591968] Walker F. (1848). List of the specimens of dipterous insects in the collection of the British Museum..

[B5592004] Westwood J. O., Westwood J. O. (1840). Order XIII. Diptera Aristotle (Antliata Fabricius. Halteriptera Clairv.). An introduction to the modern classification of insects; founded on the natural habits and corresponding organisation of the different families. Synopsis of the genera of British insects.

[B5592048] Wilkening Alan J., Kline Daniel L., Wirth Willis W. (1985). An annotated checklist of the Ceratopogonidae (Diptera) of Florida with a new synonymy. The Florida Entomologist.

[B5592038] Williston S. W. (1907). Dipterological notes. Journal of the New York Entomological Society.

[B5592067] Winnertz Joh. (1852). Beitrag zur Kenntniss der Gattung *Ceratopogon* Meigen. Linnaea Entomologica.

[B5592077] Wirth W. W. (1952). The Heleidae of California. University of California Publications in Entomology.

[B5595975] Wirth W. W. (1956). The biting midges ectoparasitic on blister beetles (Diptera, Heleidae). Proceedings of the Entomological Society of Washington.

[B5598276] Wirth W. W., Hubert A. A. (1959). Trithecoides, a new subgenus of *Culicoides* (Diptera, Ceratopogonidae).. Pacific Insects.

[B5598286] Wirth W. W., Hubert A. A. (1961). New species and records Taiwan *Culicoides* (Diptera: Ceratopogonidae). Pacific Insects.

[B5598262] Wirth W. W., Delfinado M. D., Hardy D. E. (1973). Family Ceratopogonidae. A Catalog of the Oriental Region.

[B5598296] Wirth W. W., Meillon B., Haeselbarth E., Crosskey R. W. (1980). Family Ceratopogonidae. Catalogue of the Diptera of the Afrotropical region.

[B5598310] Xue J., Liu G. P., Yu Y. X. (2003). A study of the morphological variability and description of three subspecies of *Culicoides
circumscriptus* Kieffer in different areas of China (Diptera: Ceratopogonidae).. Acta Parasitologica et Medica Entomologica Sinica.

[B5598320] Yu Y. X., Liu J. H., Liu G. P., Liu Z. J., Hao B. S., Yan G., Zhao T. S. (2005). Ceratopogonidae of China, Insecta, Diptera..

[B5598332] Zetterstedt J. W. (1850). Diptera scandinaviae disposita et descripta.

[B5598341] Zilahi-Sebess G. (1934). Beiträge zur Fliegenfauna Bulgariens. I. Chironomiden.. Bulletin de la Société Entomologique de Bulgarie.

[B5598351] Zilahi-Sebess G. (1940). Magyarorszag Heleidai. Folia Entomologica Hungarica.

